# Discovery of Anthelmintic Drug Targets and Drugs Using Chokepoints in Nematode Metabolic Pathways

**DOI:** 10.1371/journal.ppat.1003505

**Published:** 2013-08-01

**Authors:** Christina M. Taylor, Qi Wang, Bruce A. Rosa, Stanley Ching-Cheng Huang, Kerrie Powell, Tim Schedl, Edward J. Pearce, Sahar Abubucker, Makedonka Mitreva

**Affiliations:** 1 The Genome Institute, Washington University School of Medicine, St. Louis, Missouri, United States of America; 2 Department of Pathology and Immunology, Washington University School of Medicine, St. Louis, Missouri, United States of America; 3 SCYNEXIS, Inc, Research Triangle Park, North Carolina, United States of America; 4 Department of Genetics, Washington University School of Medicine, St. Louis, Missouri, United States of America; 5 Division of Infectious Diseases, Department of Internal Medicine, Genetics, Washington University School of Medicine, St. Louis, Missouri, United States of America; Sandler Center, University of California, San Francisco, United States of America

## Abstract

Parasitic roundworm infections plague more than 2 billion people (1/3 of humanity) and cause drastic losses in crops and livestock. New anthelmintic drugs are urgently needed as new drug resistance and environmental concerns arise. A “chokepoint reaction” is defined as a reaction that either consumes a unique substrate or produces a unique product. A chokepoint analysis provides a systematic method of identifying novel potential drug targets. Chokepoint enzymes were identified in the genomes of 10 nematode species, and the intersection and union of all chokepoint enzymes were found. By studying and experimentally testing available compounds known to target proteins orthologous to nematode chokepoint proteins in public databases, this study uncovers features of chokepoints that make them successful drug targets. Chemogenomic screening was performed on drug-like compounds from public drug databases to find existing compounds that target homologs of nematode chokepoints. The compounds were prioritized based on chemical properties frequently found in successful drugs and were experimentally tested using *Caenorhabditis elegans*. Several drugs that are already known anthelmintic drugs and novel candidate targets were identified. Seven of the compounds were tested in *Caenorhabditis elegans* and three yielded a detrimental phenotype. One of these three drug-like compounds, Perhexiline, also yielded a deleterious effect in *Haemonchus contortus* and *Onchocerca lienalis*, two nematodes with divergent forms of parasitism. Perhexiline, known to affect the fatty acid oxidation pathway in mammals, caused a reduction in oxygen consumption rates in *C. elegans* and genome-wide gene expression profiles provided an additional confirmation of its mode of action. Computational modeling of Perhexiline and its target provided structural insights regarding its binding mode and specificity. Our lists of prioritized drug targets and drug-like compounds have potential to expedite the discovery of new anthelmintic drugs with broad-spectrum efficacy.

## Author Summary

The World Health Organization estimates that 2.9 million people are infected with parasitic roundworms, causing high-morbidity and mortality rates, developmental delays in children, and low productivity of affected individuals. The agricultural industry experiences drastic losses in crop and livestock due to parasitic worm infections. Therefore, there is an urgent need to identify new targets and drugs to fight parasitic nematode infection. This study identified metabolic chokepoint compounds that were either produced or consumed by a single reaction and elucidated the chokepoint enzyme that drives the reaction. If the enzyme that catalyzes that reaction is blocked, a toxic build-up of a compound or lack of compound necessary for subsequent reaction will occur, potentially causing adverse effects to the parasite organism. Compounds that target some of the chokepoint enzymes were tested in *C. elegans* and several compounds showed efficacy. One drug-like compound, Perhexiline, showed efficacy in two different parasitic worms and yielded expected physiological effects, indicating that this drug-like compound may have efficacy on a pan-phylum level through the predicted mode of action. The methodology to find and prioritize metabolic chokepoint targets and prioritize compounds could be applied to other parasites.

## Introduction

Parasitic nematode (roundworm) infections impose an enormous burden of morbidity on humanity [1], [2]. Only a few drugs are commonly used to treat nematode infections, creating a dangerous environment for the emergence of drug resistance. Currently, administering anthelmintic drugs on a yearly basis is necessary to break the infection cycle, but also causes drug resistance in parasites that infect human and animal populations [3], [4]. Many of the drugs used to treat filarial infections, including diethylcarbamazine (DEC), ivermectin, and albendazole, predominately kill nematodes in their microfilarial stage and have a much lower activity level in adult worms [5]. Plant parasitic nematodes have devastating effects on crops, costing $78 billion per year globally [6]. In addition to the possibility of the development of pesticide resistance in plant parasitic nematodes, there are also environmental concerns associated with them. For example, the United States is phasing out methyl bromide (a highly effective pre-plant soil fumigant used on high-value crops) due its ability to deplete ozone in the stratosphere [7]. Thus, there is a pressing need to develop new anthelmintic treatments and pesticides [1] that are highly efficient and environmentally safe.

**Figure 1:**
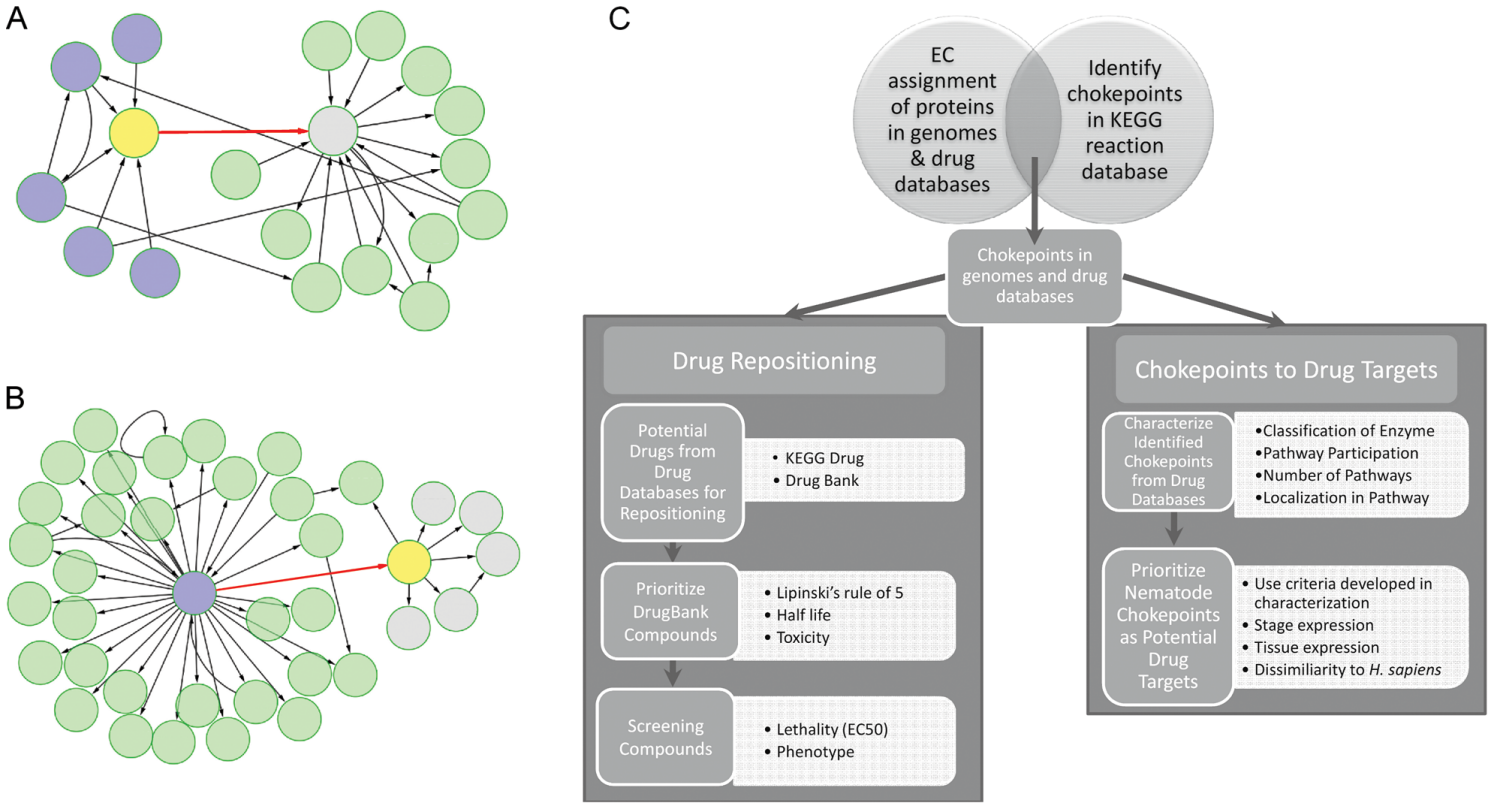
Workflow for identification, characterization, and prioritization of chokepoint drug targets and drug-like compounds. **A. & B.** The chokepoint compounds are shown in yellow. A “chokepoint reaction” either consumes a unique substrate or produces a unique product. In A., the chokepoint reaction (red) consumes a unique substrate (yellow). Five compounds are involved in reactions (blue) that produce the substrate for the chokepoint reaction. In B., the chokepoint reaction (red) produces a unique substrate, which is subsequently used in other reactions to create five new compounds (grey). **C.** Workflow diagram outlining the major steps in this study.

A systematic way of identifying new targets is by studying metabolic pathways, particularly chokepoint reactions within particular pathways. A “chokepoint reaction” is defined as a reaction that either consumes a unique substrate or produces a unique product (Figure 1A & B; [8]). If the enzyme catalyzing a reaction that produces or consumes a unique compound can be inhibited, the entire pathway will be blocked, leading to accumulation of the unique substrate or the organism being starved of unique product [8]. The idea of chokepoints and essentiality is further supported by Palumbo et al [9], which demonstrated that lethality corresponds to a lack of alternative pathways in a network that has been perturbed by a blocked enzyme.

Chokepoint analyses have been used for drug target identification in several pathogenic organisms. In two different studies, chokepoint analyses were performed to determine novel drug targets for two parasites: the mitochondrial protist, *Entamoeba histolytica* [10], and the protozoan parasite *Plasmodium falciparum*, which causes malaria [8]. Two additional studies have applied chokepoint analysis to find unique drug targets for *Pseudomonas aeruginosa* [11] (a common bacterium that causes infections) and *Bacillus anthracis* [12] (the bacterium that causes anthrax). Another study which explored *P. falciparum* drug targets has evaluated the essentiality of a reaction in a pathway by deleting a reaction *in silico* and determining if the metabolic network could find an alternative pathway to get to the same endpoint [13]. A chokepoint analysis and the essentiality of a reaction have been combined to find antibacterial drug targets [14]. However, most of these studies have yielded a long list of chokepoints without any prioritization for testing.

The number of nematodes sequenced has risen dramatically recently, with a total of 10 complete nematode genomes being published and around 30 in progress [15], [16]. These newly sequenced genomes provide a unique opportunity to find new anthelmintic drug targets that may be broad-spectrum in nature. The set of 10 sequenced nematode genomes provides representatives from four of the five clades spanning the phylum Nematoda [17] including those that are free-living, and plant, animal, or human parasitic nematodes. In this study, we determine chokepoint reactions using the intersection in all 10 nematode-deduced proteomes (the common/intersection to all ten studied nematodes, CommNem), as well as the complete set of chokepoints within the 10 deduced proteomes (the union of all 10 nematode species, UniNem). We also isolate a group of chokepoints that are only found in a union of parasitic nematodes (ParaNem). All other chokepoint analysis studies have only used a single organism in their analysis, making this pan-phylum analysis much more comprehensive than previous studies. The chokepoints from nematodes are compared to chokepoints in *Drosophila melanogaster* and *Homo sapiens*, in addition to the chokepoints found in the publicly available databases, KEGG Drug and DrugBank [18], [19]. Further, targets of insecticides were also investigated. We confirm that chokepoints are meaningful drug targets by identifying chokepoint enzymes that are already known anthelmintic and insecticide targets through this method. Given the list of nematode chokepoints, we prioritize the list by evaluating specific criteria and compare the results to known drug targets from two publically available databases. In addition, we provide a list of enzymes involved in chokepoint reactions that have already known drug associations. Seven of these compounds (referred to as “drug-like compounds” because while pharmacological properties were used to screen out compounds, not all of the compounds in those databases are approved drugs) were experimentally tested in *C. elegans* and two parasitic nematodes. Three drug-like compounds elicited a deleterious phenotype in *C. elegans*, and one of these also yielded a deleterious phenotype in the two parasitic species, demonstrating that this prioritized list of drug-like compounds should be further studied for good candidates for repositioning and/or development as potential anthelmintic drugs. We present evidence that one of these drug-like compounds, Perhexiline, acts according to its predicted mode of action. Computational modeling suggested structural differences in the binding site that can be used to develop a more specific, efficacious drug.

## Materials and Methods

### Proteomes (Deduced from Whole Genomes) Databases

The following list of nematode genomes was analyzed: *Brugia malayi* [20], *Caenorhabditi*s species from WormBase release WS240 (*Caenorhabditis brenneri, Caenorhabditis briggsae, Caenorhabditis elegans, Caenorhabditis japonicum, Caenorhabditis remanei*), *Meloidogyne hapla* (http://supfam.mrc-lmb.cam.ac.uk/SUPERFAMILY/cgi-bin/gen_list.cgi?genome=wm; [21]), *Meloidogyne incognita* (http://www.inra.fr/meloidogyne_incognita/g enomic_resources/downloads; [22]), *Pristionchus pacificus* (http://pristionchus.org; [23]) and *Trichinella spiralis* [24]. The *Homo sapiens* genome was downloaded from Ensembl (Homo_sapiens.GRCh37.57.pep.all.fa) and *Drosophilia melanogaster* were downloaded from Flybase 5.26 (http://flybase.org/static_pages/downloads/archivedata3.html). The sequences of all the genomes had open reading frames discerned and then translated to protein for analysis (henceforth referred to as ‘proteomes’). Proteins with EC (enzyme commission) numbers associated with them were downloaded from KEGG version 58 [18]. WU-BLASTP (wordmask-seg, hitdist = 40, topcomboN = 1, postsw) was used to screen the proteomes for sequence similarity and find homology to proteins with an associated EC number and best match, scoring below 1e^−10^. The intersection of ECs (i.e. common ECs, “CommNem”) and the union of ECs (i.e. set of all nematode ECs, “UniNem”) in the 10 nematode proteomes were parsed using PERL scripts developed in-house.

### Drug Databases

Both KEGG Drug [18] and DrugBank [19] were used to identify potential drugs that bind to targets in the nematode proteomes, *H. sapiens*, and *D. melanogaster*. These databases contain some FDA approved compounds, as well as compounds that were known to interact with certain targets. The KEGG Drug and DrugBank databases used for analysis were downloaded on 4/14/2010 and 5/19/2010, respectively. ECs were linking to targets using annotations from the KEGG Drug database. DrugBank contains the protein sequences of the targets, as well as their associated drugs. WU-BLASTP was used to screen the targets in DrugBank against the KEGG genes database to get an EC number annotation that matched within a cutoff score of 1e^−10^ or better. The EC number associated with the DrugBank target was then associated with the drug within DrugBank.

### Identifying Chokepoints

The reaction database from KEGG v58 [18] was used to identify chokepoint reactions and corresponding chokepoint enzymes. Each reaction equation is listed as a separate reaction with a unique identifier under the ENTRY field. The KEGG reaction database also contains a file that lists the reactions within the reaction database as reversible or irreversible (reaction_mapformula.lst – downloaded 6/21/2011). The entire reaction was extracted from the KEGG reaction database by parsing the EQUATION field, and the reaction_mapformula.lst file was used to obtain the directionality of the reaction such that the reactions could be written with reactants on the left side and products on the right side. If the reaction was reversible, this was also noted in the file because products and reactants would be ambiguous. The reactions were placed into a [compound×reaction number] matrix by parsing an intermediate file that contained the directionality and all the products and reactants for the reaction within the matrix, −1 indicated the compound was consumed (i.e. the compound was listed on the left side of the equation), +1 indicated the compound was produced (i.e. the compound was listed on the right side of the equation), 2 indicated the reaction was reversible, and a zero indicated the compound did not take part in the reaction. To find the chokepoints, the matrix was parsed for compounds that were only produced or consumed in a single reaction. If a compound was produced or consumed in a single reaction, only a single 1 or −1 would be present across the entire compound row within the matrix. In some cases, a compound was uniquely produced or uniquely consumed, but was part of a reversible reaction (i.e. two 2's would be present within a row). If this reaction was the only reaction in which the compound participated, this was also called a chokepoint. The chokepoint compounds were related to EC numbers using the ENZYME field in the reaction database.

### Pathway Participation

The EC numbers corresponding to proteins in the various genomes were mapped to KEGG metabolic pathways active in nematodes. Pathway categories that were not applicable such as photosynthesis, carbon fixation, reductive carboxylate cycle were excluded. The distribution of chokepoint targets and known drugs in metabolic pathways was compared to determine any potential enrichment using Fisher's Exact Test.

### Chokepoint Localization in Pathways

Pathways in the KEGG reaction database (v58) were enumerated. First, the KEGG reaction database was broken into separate reaction pathways based on the “PATHWAY” classification. There were 8121 entries in the reaction database, and 5638 had a PATHWAY classification. Only 142 unique reaction pathways were used; due to the large size and overlap with other pathways, rn00240, rn00230, rn01100, rn01110, and rn01120 were not used. For each of the different pathways, a separate [compound×reaction number] matrix was generated as described in the “Identifying Chokepoints” section above. The starting and ending nodes for reaction pathways were generated from this matrix by determining compounds that were consumed but not produced (start nodes) and produced but not consumed (end nodes). Beginning with each of the start nodes, the compounds in all possible pathways were enumerated. The position of the chokepoint within the pathway was determined by the number of compounds in the pathway before the chokepoint, as well as the length of the entire pathway.

### Prioritization of Chokepoint Reactions and Targets

Chokepoint enzymes were prioritized by assigning a point for meeting each of the following criteria, then ranked based on number of points: EST-based gene expression found in a parasitic stage for plant parasitic nematodes (egg, J2, J3, J4, adult) and infective/parasitic stages for human and animal parasitic nematodes (embryo, L3, L4, adults); expressed in pharynx, intestine, neurons, muscle, or hypodermis [25], [26], [27] in *C. elegans* (www.wormbase.org); less than 30% sequence identity to *H. sapiens* over half the length of the sequence; chokepoint enzyme functioning in two or more pathways; chokepoint enzyme involved in nucleic acid metabolism; and chokepoint is a hydrolase based on their enrichment (classification as EC 3, enzyme commission number). This analysis was performed to determine if certain classes of enzymes were more likely to have drugs associated with them. This information was fed into the prioritization scheme. EST sequences sets for the 10 species were downloaded from Genbank on 7/16/2010: *C. brenneri*, *C. briggsae*, *C. japonicum*, *M. hapla*, *M. incognita*, *T. spiralis*, *P. pacificus*, *B. malayi*, and *C. remanei*. *C. elegans* EST sequences were downloaded from GenBank on 4/21/2010. The tissue expression data from *C. elegans* was obtained from WormMart (WS195) on 4/23/2010.

### Chemogenomic Screening for Compound Prioritization

Proteins associated with ECs (using KEGG) were blast searched against protein targets in DrugBank as described above. The ECs from DrugBank were compared to CommNem and UniNem. Cheminformatic properties were obtained by running SMILES strings (SMILES are strings of ASCII characters that describe a compound unambiguously) extracted from DrugBank through the Cytoscape [28] plugin, ChemViz. To prioritize the drugs, drugs were given one point for meeting each of the following criteria: molecular weight ≤500, 0<number of rotatable bonds ≤10, hydrogen-bond donors ≤5, hydrogen-bond acceptors ≤10, logP≤5 [29]. This additional screen was done because the compounds in the drug database are not optimized for Lipinski's rules and thus may not have been “successful” drugs for the disease for which they were developed/tested. For a drug to be effective, it should have a long half-life, so a drug with half-life ≥60 minutes was rewarded with a point. Toxicity information is also important for future testing and therefore, a compound with any available toxicity information was given an additional point. The maximum attainable compound score was 7. Drug-like compounds were also eliminated if placed in the dietary supplement, micronutrient, or vitamin categories by DrugBank, as various vitamins and amino acids were not desired. Nematode proteins were searched against sequences from DrugBank, and then parsed for sequences that had 50% or greater identity over 80% of sequence length. Only these targets were considered in the prioritized list.

### Compound Screen in *Caenorhabditis elegans*

Compounds were obtained from the following sources: Perhexiline maleate (**1** DB1074 is just perhexiline; CAS: 6724-53-4; P287320) from TRC; Carbidopa (**2** DB00190; CAS: 28860-95-9; BML-EI265) and dopamine (**4** DB00988; CAS: 62-37-1; BML-AC752) were ordered from Enzo Life Sciences dissolved in DMSO; LT00772250 (Probenecid 5 DB01032; CAS: 57-66-9), LT00255846 **3** (similar to DB00993; the DrugBank compound was not available, so a similar compound was ordered), LT00138053 (**6** DB01033), LTBB001666 (**7** DB00548) were ordered from Ryan Scientific. Compounds formulated in 100% DMSO were tested in microtiter plates containing 50 µl nematode growth media, 1% *E. coli* and 20 L1 *C. elegans*. Five concentrations in 4-fold increments (0.078, 0.3125, 1.25, 5, and 20 ppm; ∼25 to 60 µM, depending on the molecular weight of the compound) were tested, and the experiment was repeated twice and a final confirmation test, with the best result reported. The efficacy of a compound was determined based on the motility of the larvae as compared to average motility of control wells containing DMSO only at 48 hours post treatment (by that time the larvae develop to L4's; screening is not performed at a later stage due to the way imaging is done, i.e. comparing exact numbers of parasites in every well). The motility was assessed using a camera-based imaging. The camera takes multiple images of a well and the changes in movement between the images are calculated. An absolute movement value is calculated for each well. On each test plate, multiple wells containing only DMSO are included as a control. The absolute movement value from these wells was averaged and then compared to the movement in the treatment wells. The percent reduction in motility is calculated by dividing the movement in the treatment well by the average movement of the DMSO wells. Controls were used on every plate and in every test (data not shown). Movement was manually assessed at 72 hours post-treatment to determine if there were altered movements or morphological changes not detected by the imaging system.

### Compound Screen in Parasitic Nematodes

Compounds formulated in 100% DMSO were tested in microtiter plates containing 50 µl nematode media, fecal slurry and 20 L1 *Haemonchus contortus*. The experiment was repeated twice at five concentrations in 4-fold increments (0.078, 0.3125, 1.25, 5, and 20 µM). The efficacy of a compound was determined based on the motility of the larvae (when the larvae have developed to L3's) as compared to average motility of control wells containing DMSO only. A MIC90 value was calculated by determining the lowest dose at which there was a 90% reduction in motility as compared to the control wells. The motility was assessed using a camera-based imaging system as described in the *C. elegans* screen. Larval movement was manually assessed at 72 hours post-treatment to determine if there were altered movements or morphological changes not detected by the camera.

Compounds were tested at two static doses of 50 µM and 12.5 µM in *Onchocerca lienalis*. Five microfilariae were added to each well of a 96-well microtitre plate. Larvae were assessed at 120 hours post-treatment and efficacy was determined by visually assessing the motility of the larvae in the treated wells as compared to control wells.

While other stages for screening could also be used, our approach was implemented as an early indicator of activity. Progressing to advanced tests against relevant clinical stages should be the next step for future research. In particular, when working with filarial worms, having some filter for prioritizing compounds is helpful, since access to adult stages is often difficult.

### Measurement of Oxygen Consumption Rates

Real-time measurements of oxygen consumption rates (OCR) were made using an XF-24 Extracellular Flux Analyzer (Seahorse Bioscience) as previously described [30]. The real-time extracellular flux experiment was designed to evaluate whether Perhexiline decreases OCR via inhibiting mitochondrial carnitine palmitoyltransferase in *C. elegans*. The concentrations used (25–100 uM) do not have any impact on the movement of the worms (based on examination under the microscope), but do have an impact on the OCR. Synchronized young adult *C. elegans* were washed with M9 media and plated into XF-24 culture plates at approximately 100 worms/well. OCR measurements were recorded under basal conditions or in the presence of Perhexiline, Etomoxir (Sigma) and/or Ivermectin (Sigma) at various concentrations, over a period of 1.5 hours and 40 minutes. The significance of observed OCR differences was assessed using Student's *t*-test using GraphPad Prism Version 5.

### RNA Extraction and RNAseq Data Generation

The treated worms (approximately 100 µl settled volume) were washed in sterile PBS and resuspended in 100 µl TRIzol reagent (Invitrogen). Samples were frozen with liquid nitrogen and homogenized. Following the homogenization, the worm/TRIzol powder was collected and allowed to thaw on ice. A further 0.2 volumes of chloroform were added into samples, and gently mixed, incubated at room temperature for 3 minutes, then centrifugated at 12,000× *g* for 15 minutes at 4°C. The upper aqueous phase was transferred to a fresh tube and RNA was precipitated by an additional 0.5 volumes of isopropanol followed by incubation at room temperature for 10 minutes. The mixture was then centrifuged at 12,000× g for 10 minutes at 4°C. The supernatant was discarded and the RNA pellet was washed with 500 µl of 75% (v/v) ethanol before centrifugation at 7,500× *g* for 5 minutes at 4°C. The supernatant was removed and the pellet air-dried. The RNA pellet was suspended in nuclease-free distilled water.

The total RNA was treated with Ambion Turbo DNase (Ambion/Applied Biosystems, Austin, TX). 1 ug of the DNAse treated total RNA went through polyA selection via the MicroPoly(A) Purist Kit according to the manufacturer's recommendations (Ambion/Applied Biosystems, Austin, TX). 1 ng of the mRNA isolated was used as the template for cDNA library construction using the Ovation RNA-Seq version 2 kit according to the manufacturer's recommendations (NuGEN Technologies, Inc., San Carlos, CA). Non-normalized cDNA was used to construct Multiplexed Illumina paired end small fragment libraries according to the manufacturer's recommendations (Illumina Inc, San Diego, CA), with the following exceptions: 1) 500 ng of cDNA was sheared using a Covaris S220 DNA Sonicator (Covaris, INC. Woburn, MA) to a size range between 200–400 bp. 2) Eight PCR reactions were amplified to enrich for proper adaptor ligated fragments and properly index the libraries. 3) The final size selection of the library was achieved by an AMPure paramagnetic bead (Agencourt, Beckman Coulter Genomics, Beverly, MA) cleanup targeting 300–500 bp. The concentration of the library was accurately determined through qPCR according to the manufacturer's protocol (Kapa Biosystems, Inc, Woburn, MA) to produce cluster counts appropriate for the Illumina platform. The HiSeq2000 Illumina platform was used to generate 100 bp sequences.

### Analytical Processing of the Reads and Differential Expression

Analytical processing of the Illumina short-reads was performed using in-house scripts. DUST was used to filter out regions of low compositional complexity and to convert them into N's [31]. An in-house script was used to remove N's, which discards reads without at least 60 bases of non-N sequence. Raw RNA-seq datasets are deposited at SRA (accession numbers: Control - SRR868958, IVM - SRR868932, PER - SRR868957, PER+ETO - SRS868939, ETO - SRS868942.). Gene expression for each sample was calculated by mapping the screened RNA-seq reads to the WS230 release of *C. elegans* using Tophat [32] (version 1.3.1), and calculating depth and breadth of coverage per gene using Refcov (version 0.3, http://gmt.genome.wustl.edu/gmt-refcov). Gene expression values were normalized using the depth of coverage per million reads (DCPM) per sample [33]. Expressed genes were subject to further differential expression analysis using EdgeR [34] (false discovery rate <0.05, dispersion value 0.01), in order to identify genes differentially expressed in each treatment relative to the control sample. Hierarchical agglomerative clustering (with “unweighted pair group method with arithmetic mean”, and Pearson correlation coefficient similarity settings in XLSTAT-Pro; version 2012.6.02, Addinsoft, Inc., Brooklyn, NY, USA) was used to cluster samples based on the gene expression profiles across all genes, and to cluster all 1,908 genes upregulated in any of the four comparisons.

### Functional Annotation and Enrichment

Interproscan [35], [36] was used to determine associations of genes to Gene Ontology (GO) terms [37]. Interproscan also identified predicted Interpro domains found in each gene. GO term enrichment among genes upregulated in each of the 4 samples was determined using a non-parametric binomial distribution test with a 0.05 p value cutoff for significance, after Benjamini-Hochberg false-discovery-rate (FDR) population correction for the total number of terms [38]. Only GO terms with at least 5 gene members in the *C. elegans* genome were included in the analysis (501 total).

### Docking Perhexiline

Perhexiline was downloaded from the DrugBank website as a mol file, then converted to a PDB file using OpenBabel [39]. The PDB file was optimized using Sybyl 7.3 [40] to minimize the Perhexiline structure. In AutoDockTools4 [41], hydrogen atoms, followed by Gasteiger charges, were added, then the non-polar hydrogen atoms were merged. A docking box of 88×68×80 points in the x, y, and z dimensions, with a spacing of 0.375 Å, was used centered at 61.752, 72.8001, 52.0321 and all other parameters were default. The carnitine palmitoyltransferase-2 (CPT-2) macromolecule was taken from the crystal structure of 2H4T [42]. Hydrogen atoms were added, followed by Kollman charges. Then, the non-polar hydrogens were merged on the macromolecule. The docking calculations utilized local search Lamarkian genetic algorithm in Autodock4 [41] using rigid side chains. A total of 250 genetic algorithm runs were done. The results were clustered using Autodock4 with the default parameters.

## Results

### Identification of Chokepoint Enzymes and Their Phylogenetic Distribution

**Figure 2:**
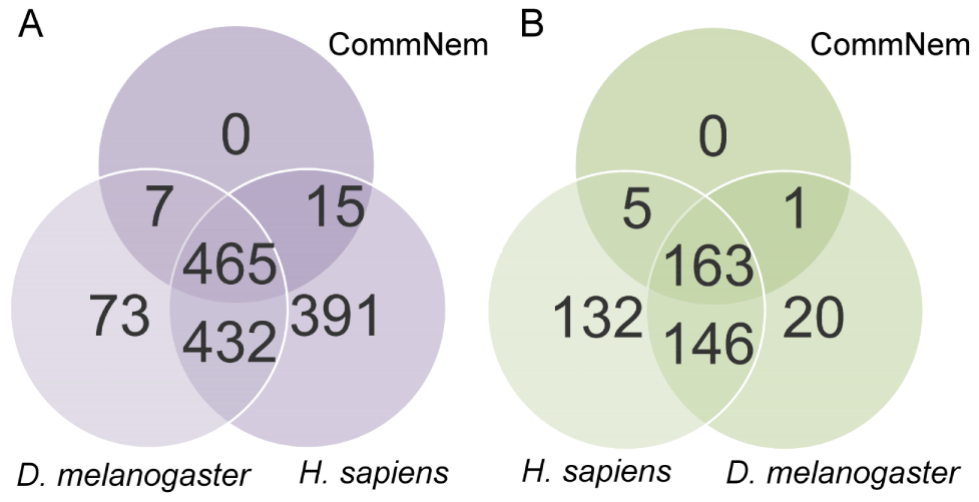
Proteins with Enzyme Commission (EC) classification and chokepoint enzyme mapping. Intersection of **A.** Proteins with EC classification and **B.** Chokepoint enzymes from CommNem, *D. melanogaster*, and *H. sapiens*. The 487 proteins that are referred to in the text from CommNem are derived from Figure 2A (7+465+15), and the 169 proteins from CommNem that are conserved chokepoints are derived from Figure 2B (5+163+1).

Our approach identifies chokepoint enzymes as targets of existing drugs or as novel drug targets (Figure 1C). The intersection of nematode genomes (CommNem) yielded 487 proteins conserved among all nematode species studied, of which 169 are conserved chokepoint enzymes (Figure 2 & Table S1 in Text S1). The union of the nematode proteomes (UniNem) yielded 477 chokepoint enzymes (Table S2 in Text S1), of which 24 chokepoint enzymes were only found in parasitic worms (ParaNem). The EC numbers and corresponding FASTA sequences for each of the species investigated can be found on Nematode.net [43]. In all cases, 34–35% of the proteome assigned with an EC number consists of chokepoints (Figure S1 in Text S2). The only chokepoint enzyme present in CommNem and not in *H. sapiens* is EC: 6.2.1.12. However, 120 chokepoint enzymes from UniNem are not found in *H. sapiens*. A high overlap also exists between CommNem chokepoint enzymes and *D. melanogaster*, with only 5 of 169 in CommNem are not present in *D. melanogaster* (EC: 1.8.4.2, 2.4.2.8, 5.3.2.1, 2.7.1.149, 3.6.1.14).

### Chokepoint Enzyme Classification

Some enzyme categories were enriched or depleted based on Fisher's Exact statistical test within the species relative to chokepoint enzymes in KEGG (i.e. KEGGChoke), and all enzymes in the KEGG database (i.e. AllKEGG) (Figure S2 in Text S2). This analysis was performed to determine if certain types of enzymes were more likely to have drugs associated with them. This information was fed into the prioritization scheme. Oxidoreductases were significantly enriched in nematodes and KEGG Drug and DrugBank relative to KEGGChoke (p<0.005). The chokepoints within KEGG Drug and DrugBank were significantly enriched in hydrolase enzymes (p<0.005) when compared to KEGGChoke (all chokepoints in KEGG identified using our approach) as well as AllKEGG (all enzymes with assigned ECs within KEGG). Further, isomerases in DrugBank and KEGG Drug were significantly enriched relative to KEGGChoke. The abundances of enzymes in DrugBank and KEGG Drug significantly differ from KEGGChoke in 3 out of the 6 enzyme categories.

### Anthelminth Chokepoints and Chemogenomic Screening

There are 75 drugs in KEGG Drug that are classified as anthelmintic. Much research has also been done to design insecticides, therefore it is interesting to see that these insecticides also target chokepoint enzymes. The insecticides are shown in Table S3 in Text S1, and the DrugBank compounds that are classified as antiparasitic are shown in Table S4 in Text S1.

The nearly complete overlap of CommNem and partial overlap of UniNem chokepoint enzymes with *H. sapiens* enzymes provide an excellent opportunity to reposition drugs used for other purposes in *H. sapiens* as anthelmintic drugs. If these drugs show some efficacy, subsequent optimization studies could be performed on these leads to make these drugs bind with higher affinity and specificity to the nematode protein. Out of the 169 chokepoints in CommNem, only 13 have a drug associated with them in KEGG Drug (Table S5 in Text S1 and Table S6 in Text S1). When considering UniNem, a total of 29 chokepoints have ECs associated with a drug in KEGG Drug (Table S5 in Text S1 and Table S7 in Text S1). Out of 446 enzymes involved in chokepoint reactions in *H. sapiens*, only 35 mapped to ECs associated with a drug in KEGG (data not shown). Of the 977 enzymes in the *D. melanogaster* genome, 330 are chokepoint enzymes and of the 68 of those that mapped to the ECs in the KEGG Drug database 29 are considered chokepoint enzymes. There are 30 drugs in KEGG that have insecticide activity, but none have ECs associated with them. Only 97 enzymes within KEGG Drug have an EC assigned, of which 39 are associated with chokepoint reactions. Therefore, the UniNem, *H. sapiens*, and *D. melanogaster* proteins hit roughly 1/3 of targets with ECs assigned within KEGG Drug.

**Figure 3:**
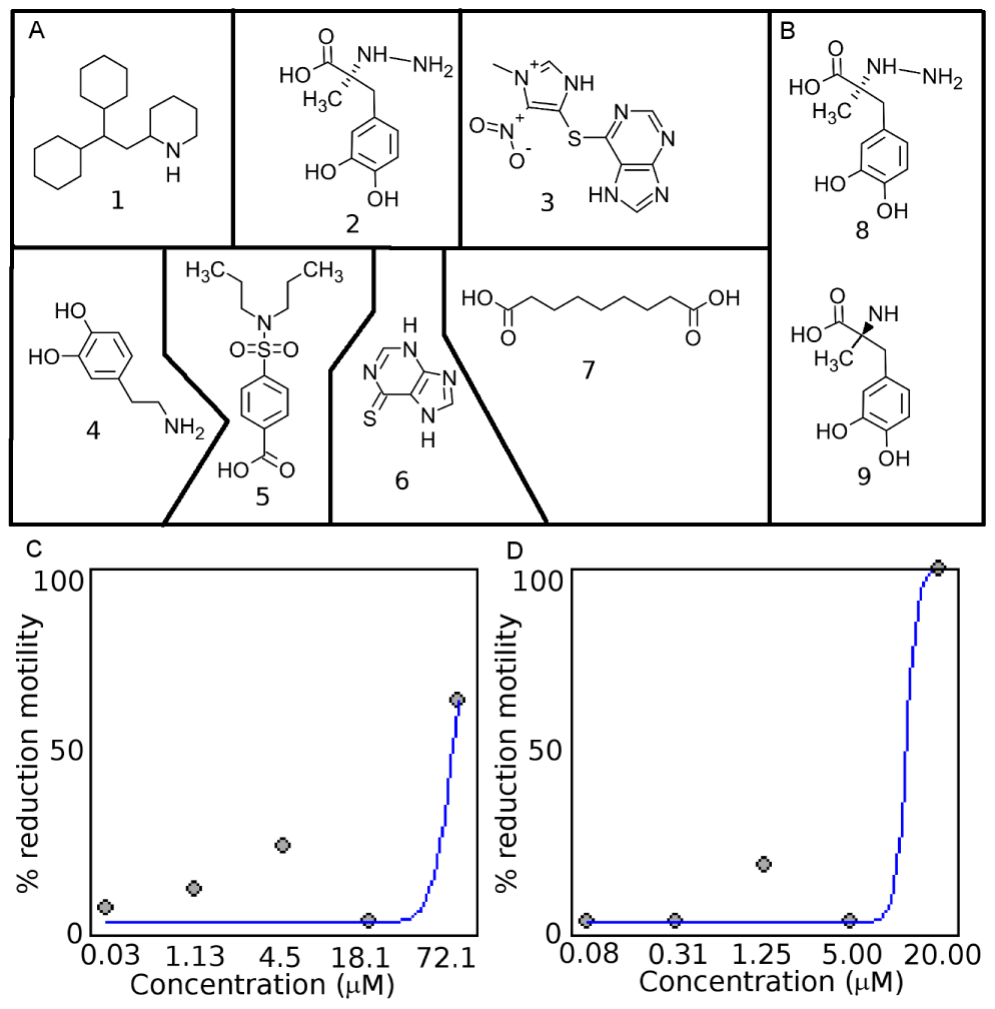
Chemical structures of drug-like compounds and results from screening in *C. elegans* and *H. contortus*. **A.** The drug-like compounds tested in the *C. elegans*, *H. contortus*, and *O. lienalis* screens. **1** (perhexiline; DB01074), **2** (DB00190), **3** (LT00255846 - DB00993 is a similar compound), **4** (DB00988), **5** (DB01032), **6** (DB01033), **7** (DB00548). **B.** Comparison of **8** (DB00190) from this study and **9** (Methyldopa), which was shown to inhibit L-DOPA decarboylase in *S. mansoni*. Dose-response curve for perhexiline (**1**) in **C.** *C. elegans* and **D.** *H. contortus*.

DrugBank contains the sequences of targets to which the drugs bind, enabling more complete mapping of ECs to protein targets and subsequently to drug-like compounds. Within DrugBank, there are 4774 compounds, and 1289 targets were assigned EC numbers. DrugBank contains 504 enzymes that are involved in chokepoint reactions based on chokepoints derived from KEGG reactions. Based on the number of compounds, KEGG Drug has more compounds than DrugBank with 9447 compounds. However, DrugBank has many more compounds associated with ECs (Figure S1 in Text S2). Due to the large list of targets and compounds, the compounds were prioritized (*see* Methods). Several of the compounds yielded the maximal compound score of 7. A compound score cutoff of ≥6 was used to prioritize the top drugs that have potential to be repositioned or further optimized as nematode drugs (Figure 3A, Table 1). The compounds identified are drugs that are used to treat hypertension, angina, and Parkinson's disease, and have immunosuppressive and antimicrobial properties.

**Table 1:** Prioritized list of drug-like compounds from DrugBank that can potentially be repositioned or further optimized for nematodes.

Comp #	DB ID (Name)	CompScore^a^	EC	Group^b^	Description^c^	Ce Results	Hc & Ol Results^d^
**1**	DB01074 **(Perhexiline)**	7	2.3.1.21	Comm Nem	Treatment of angina; Increases glucose metabolism at the expense of fatty acid met.	EC50 = 47.3 µM	+
**2**	DB00190 **(Carbidopa)**	7	4.1.1.28	Comm Nem	Inhibits DOPA decarboxylase. Treatment for Parkinson's disease	+	−
**3**	DB00993^e^ **(Azathioprine)**	7	4.3.2.2	Comm Nem	Immunosuppressive drug to treat rheumatoid arthritis	−	−
**4**	DB00988 **(Dopamine)**	6	1.14.17.1	Comm Nem	Dopamine	−	−
**5**	DB01032 **(Probenecid)**	6	6.1.1.9	Comm Nem	Inhibits renal excretion; used as adjunct to antibacterial therapy	−	−
**6**	DB01033 **(Mercaptopurine)**	6	4.3.2.2	Comm Nem	Antineoplastic with immunosuppressant properties; inhibits purine metabolism	−	−
**7**	DB00548 **(Azelaic Acid)**	7	1.3.99.5	Uni Nem	Antimicrobial for acne	+	−

a See Methods;

b All CommNem targets are a subset of UniNem.

c The descriptions were taken from DrugBank.

d Ce = *C. elegans*, Hc = *H. contortus*, Ol = *O. lienalis*; + indicates an observed phenotype; − indicates no observed phenotype.

e Similar compound was tested experimentally.

### Chokepoint Characterization

#### Pathway population

Various groups of enzymes involved in chokepoint reactions (including UniNem, CommNem, KEGG Drug, and DrugBank) were compared to KEGGChoke and AllKEGG. The enzymes in CommNem were involved in a significantly greater number of multiple pathways than AllKEGG and KEGGChoke (p<10^−4^) (Figure S3A in Text S2). For the drug databases, significantly fewer enzymes in KEGG Drug and DrugBank were involved in just one pathway and significantly more were involved in multiple pathways when compared to KEGGChoke (p<0.02; Figure S3B in Text S2). When KEGG Drug and DrugBank were compared to AllKEGG, there were significantly fewer enzymes that were involved in just one pathway (p<0.03). The information obtained from these tests was added to the prioritization scheme, providing an additional point (resulting in a higher score) for chokepoint enzyme involved in two or more pathways due to higher likelihood to have a deleterious effect when inhibited.

**Figure 4:**
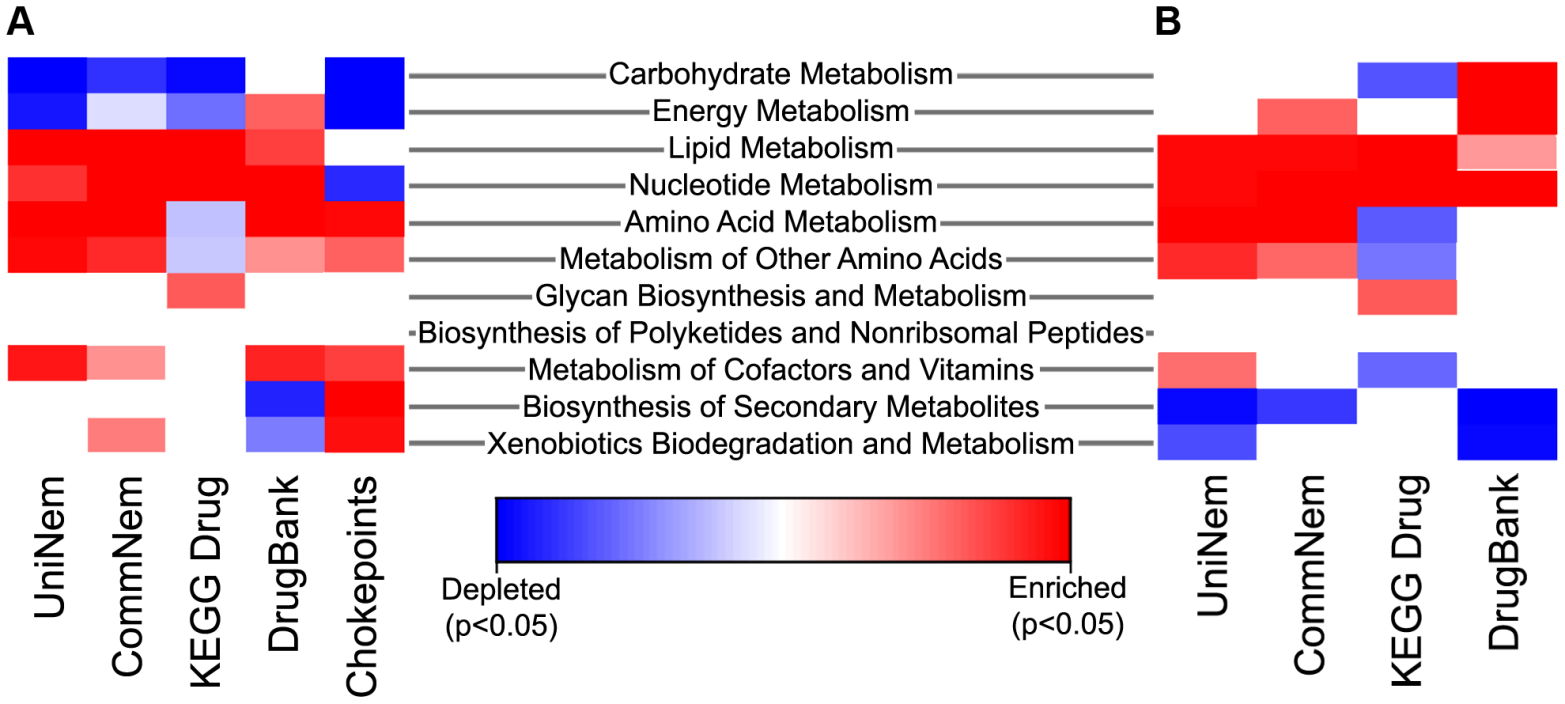
Heatmap indicating enriched and depleted KEGG metabolic pathways. The extreme blue color indicates that the enzyme category was significantly depleted and the extreme red color indicates the enzyme category was significantly enriched relative to either all chokepoint enzymes or all the EC values from KEGG using Fisher's Exact Test. The intermediate color shades indicate enrichment or depletion, but are not statistically significant. Enrichment or depletion of metabolic pathways in UniNem, CommNem, DrugBank, and KEGG Drug compared to **A.** AllKEGG and **B.** KEGGChoke. CommNem, intersection of nematode ECs; UniNem, set of all nematode ECs; KEGGChoke, chokepoint enzymes within KEGG; AllKEGG, all enzymes within KEGG; DrugBank, ECs from DrugBank; KEGG Drug, ECs from KEGG Drug.

Chokepoint enzymes and potential drug targets may be enriched in certain metabolic pathways. Where nematode-appropriate pathways were considered (excluding pathways that are not applicable, such as photosynthesis, etc, since the KEGG pathways are a common set of pathways for prokaryotes and eukaryotes), nucleotide metabolism was enriched significantly in UniNem, CommNem, KEGG Drug, and DrugBank when compared KEGGChoke (Figure 4). The enzymes in DrugBank were enriched in several areas of nematode metabolism, including nucleotide, energy, and carbohydrate metabolism, and depleted in biosynthesis of secondary metabolism and xenobiotics biodegradation compared to KEGGChoke. KEGG Drug was enriched in two areas of metabolism: lipid and nucleotide compared to KEGGChoke. CommNem was also enriched in enzymes involved in amino acid metabolism when compared to KEGGChoke. When the groups were compared to AllKEGG, enrichment for UniNem included lipid metabolism, amino acid metabolism, and metabolism of other amino acids. Enzymes with nucleotide and amino acid metabolism were also significantly enriched in the CommNem group compared to AllKEGG.

#### Position in pathway

The chokepoint position within their respective pathway was calculated by taking the position in the pathway divided by the total pathway length. The pathways ranged in length from 3 to 36 reactions, with most pathways within one standard deviation of the mean (15 reactions) being 10 to 21 reactions long. The positions of the chokepoint compounds in the pathways were relatively evenly distributed throughout the pathway in *H. sapiens*, *D. melanogaster*, and CommNem, regardless of whether the compound was produced or consumed. The position of chokepoint enzymes that already have known drugs associated with them were mapped to their position in the pathways. Chokepoint enzymes whose product were consumed were enriched (using Fisher's Exact Test) around the point of 20% pathway length for anthelmintic compounds (p<2e^−8^) compared to ∼60–70% of the pathway length for chokepoints in KEGG Drug (p<2.2e^−16^). Chokepoint enzymes whose compound was created were enriched (using Fisher's Exact Test) around the point of 50% pathway length for anthelmintic compounds (p<4e^−15^) compared to around 70% for compounds in KEGG Drug (p<2.2e^−16^). This knowledge was not added to the prioritization, however, because significant results were not obtained for chokepoint enzymes in DrugBank. Additional testing would need to be done on more drug databases before this could be incorporated into prioritization.

### Prioritization of Chokepoint Enzymes and Experimental Testing

The chokepoint enzymes were prioritized for the CommNem, UniNem, and ParaNem groups using a simple addition scoring function, with 7 being the maximum possible target score (*see* Methods and Materials). The results for CommNem and UniNem are shown in Table 2 and ParaNem in Table S8 in Text S1. The maximum target score obtained in CommNem and UniNem was 5, and a cutoff of 4 was used. None of the enzymes in ParaNem met the maximum-target score criteria as well, with 5 being the highest target score attained; therefore a cutoff of 2 was used.

**Table 2:** Top prioritized chokepoint enzymes.

Group^a^	EC number	Target score	Name	Type of enzyme	Previous Indications as Drug Target
CommNem	3.5.2.2	5	dihydropyrimidinase	Hydrolase	Tumor suppressor target [68]
	2.7.1.40	4	pyruvate kinase	Transferase	Drug target for *P. falciparium* [69] and bacteria [70]
	2.7.4.6	4	nucleoside-diphosphate kinase	Transferase	Some are secreted in *T. spiralis* and may modulate host cell function; Found to be consist. Trans. During all parasitic stages in *B. malayi*. Did molecular modelling for drug targeting [58]
	2.7.7.4	4	sulfate adenylyltransferase	Transferase	----------------------
	3.1.3.11	4	fructose-bisphosphatase	Hydrolase	Drug target for Type 2 diabetes [71]
	3.1.3.5	4	5′-nucleotidase	Hydrolase	Clinical vs Environmental isolates *B. cepacia* – secretion higher in clinical – might be way bacteria evades immune system [72]; Inhibited by plant compounds lycorine and candimine in *T. vaginalis* [55]; Inhibitors to treat melanomas, gliomas, breast cancer, gastrointestinal infections and bacterial diarrhea, and hepatic fibrosis
	3.1.4.17	4	3′,5′-cyclic-nucleotide phosphodiesterase	Hydrolase	Drug target for *P. falciparum* [73] and kinetoplastids [74]; psychiatric and neurodegenerative diseases [75]
	3.2.1.52	4	beta-N-acetylhexosaminidase	Hydrolase	Involved in chitin remodeling and drug target in *T. vaginalis* [76]
	3.5.4.5	4	cytidine deaminase	Hydrolase	Drug target for *M. tuberculosis* [77]; Anticancer therapeutic potential [78]
	3.5.5.1	4	Nitrilase	Hydrolase	Tumor suppressor target [79]
	3.6.1.19	4	nucleoside-triphosphate diphosphatase	Hydrolase	Inhibited by plant compounds lycorine and candimine in *T. vaginalis* [55]; New class of antischistosoma drugs partially inhibit suggesting that inhibition may negatively effect survival.
	3.6.1.29	4	bis(5′-adenosyl)-triphosphatase	Hydrolase	----------------------
UniNem	3.1.3.1	4	alkaline phosphatase	Hydrolase	----------------------
	3.2.1.26	4	beta-fructofuranosidase	Hydrolase	----------------------
	3.5.1.6	4	β-ureidopropionase	Hydrolase	----------------------
	3.7.1.2	4	fumarylacetoacetase	Hydrolase	Target for treating tyrosinemia [80]

a All CommNem targets are a subset of UniNem.

### Drug-like Compound Screening in *C. elegans*, *H. contortus*, and *O. lienalis*

The seven drug-like compounds prioritized based on our cut-off (*see* Methods and Materials) were experimentally screened in *C. elegans* (Table 1), and three yielded a phenotype. *C. elegans* exposed to drug-like compound **2** yielded a slow moving and twitchy phenotype, whereas **7** yielded a jerky, twitchy phenotype in 75% of the worms and 25% of the worms did not move after exposure to the compound. *C. elegans* exposed to drug-like compound **1** (Perhexiline) yielded a 50% reduction in motility phenotype at 47.3 µM (18.6 ppm), also showed slow movement and twitchy behavior at compound concentrations below the EC50 value. Importantly, Perhexiline (**1**) caused a 90% reduction in motility (MIC90) at 20 µM in the blood-feeding nematode *H. contortus*, and 100% reduction in motility in the filarial nematode *O. lienalis* at 50 µM. Chemical structures of the drug-like compounds are shown in Figure 3A, dose-response curves for Perhexiline (**1**) are shown in Figure 3C & D, and videos of the effect of Perhexiline (**1**) on *C. elegans* and *H. contortus* and Carbidopa (**2**) and Azelaic acid (**7**) in *C. elegans* are shown in Supplementary Videos (Video S1, S2, S3, S4, S5, S6, S7, S8).

### Measurement of Oxygen Consumption Rates

**Figure 5:**
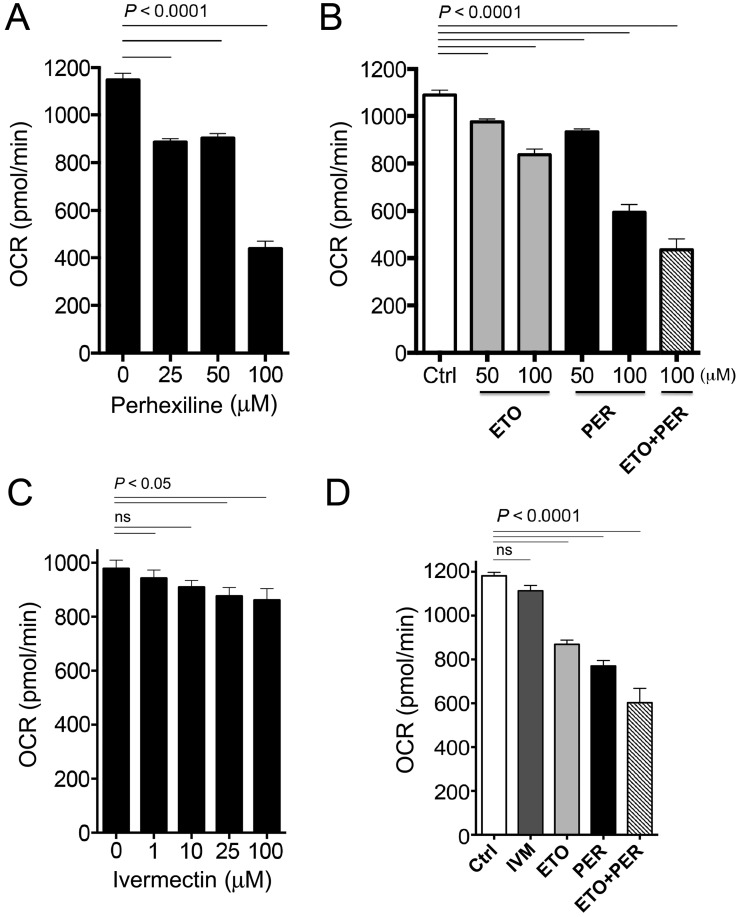
Oxygen consumption rates in *C. elegans* after exposure to varying concentrations of Perhexiline (PER), Etomoxir (ETO) and Ivermectin (IVM). **A.** Average basal oxygen consumption rates (OCR) of adult *C. elegans* incubated with vehicle (1% DMSO, 0 µM) or different concentrations of PER (CPT-2 inhibitor) (25, 50 and 100 µM) over 90 minutes. **B.** Average OCR of adult *C. elegans* incubated with vehicle (1% DMSO, Ctrl), ETO (CPT-1 inhibitor) (ETO, 50 and 100 µM), PER (50 and 100 µM), or 100 µM ETO plus 100 µM PER (ETO+PER) over 90 minutes. **C.** Average based OCR of adult *C. elegans* incubated with vehicle (1% DMSO, 0 µM) or different concentrations of IVM (binds to glutamate-gated chloride channels) over 40 minutes. **D.** Average OCR of adult *C. elegans* incubated with vehicle (1% DMSO, Ctrl), IVM (10 µM), PER (100 µM), ETO (100 µM), or 100 µM PER+100 µM ETO, over 40 minutes. Data are representative of at least 2 individual experiments. Bars represent the ±SEM of 15 OCR readings from 4 independent replicates per experiment. The experiment was repeated twice.

Carnitine palmitoyl transferases (CPT) are chokepoint enzymes with existing drugs, such as Perhexiline (**1**), inhibiting the mammalian homologs. Two versions of the enzyme (CPT-1 and CPT-2) play important roles in fatty acid metabolism in the mitochondria [44]. Inhibition of CPT leads to a decrease in oxygen consumption rate (OCR) in the mitochondria. Perhexiline (drug-like compound **1**) treatment in *C. elegans* led to a significant decrease in basal OCR in a dose-dependent manner (Figure 5A). The effect of Perhexiline (PER) was equivalent to that of Etomoxir (ETO), a known inhibitor of the mitochondrial outer membrane associated enzyme, CPT-1, which acts with CPT-2 to regulate fatty acid oxidation [45], [46]. The combination of Perhexiline and Etomoxir had an additive inhibitory effect of OCR that was greater than the effects measured with either drug alone (Figure 5B).

OCR was also measured in presence of PER, ETO, PER+ETO and compared to OCR in presence of Ivermectin (IVM), a commercially available anthelminic used to treat nematode infections. IVM, which kills *C. elegans* at therapeutic concentrations through interference with nervous system function, provides a control for drug-induced toxicity that leads to phenotypic alterations such as paralysis that may indirectly affect oxygen consumption as measured by OCR. The dose response curve (Figure 5C) enabled identification of the 10 uM concentration as applicable for our comparison experiment (see Methods). While the effect of PER, ETO and the additive inhibitory effect of OCR was confirmed by this experiment, the IVM had no significant inhibitory effect of OCR (Figure 5D).

### Gene Expression Profiles of *C. elegans* after Exposure to PER, ETO and IVM

**Figure 6:**
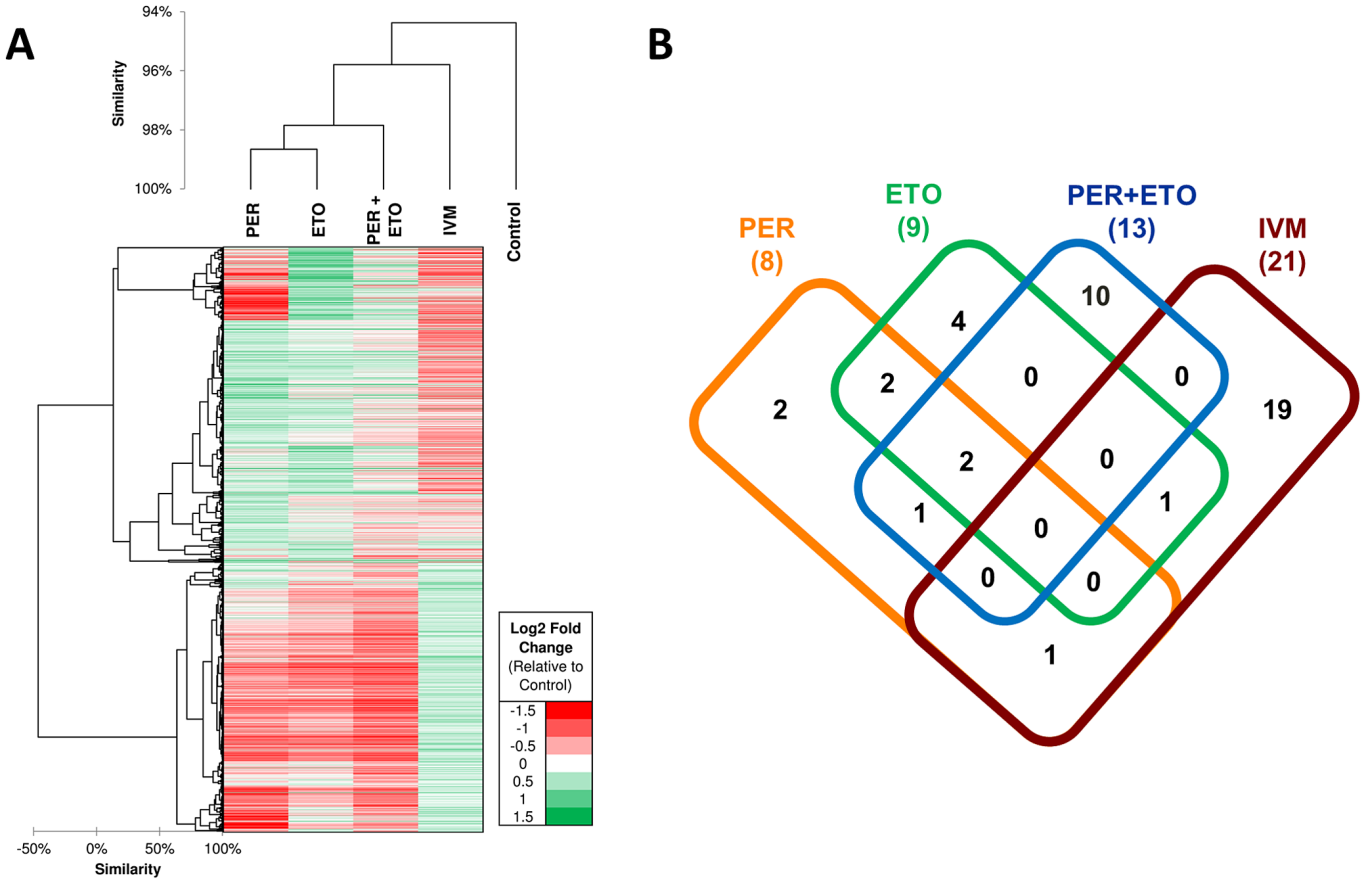
Transcriptional response of *C. elegans* in the presence of Perhexiline (PER), Etomoxir (ETO) and Ivermectin (IVM). **A.** Hierarchical clustering of samples based on gene expression patterns across all genes, and a heatmap based on differential expression profiles of 1,908 genes which were upregulated in at least one of the four samples relative to the control. **B.** Distribution of Gene Ontology enriched categories among the upregulated genes in each of the four samples. The list of GO categories is provided as Table S10.

Genome-wide gene expression profiling can be used to investigate if a transcriptional response to drugs carries signatures for drug mechanism of action. Drugs with related mechanisms of action are expected to have similar patterns of molecular functions significantly perturbed. RNAseq-based expression evidence was obtained for all *C. elegans* genes with 6–11% of the genes being differentially expressed among the four treatments (Table S9 in Text S1). On average 2–8% of genes were upregulated (range 1.7% PER+ETO to 3.9% IVM) and 5.3% were downregulated (range 3.3% PER to 7.1% IVM). Comparison of genome-wide transcriptional responses to PER, ETO, PER+ETO and IVM showed that the transcriptional responses of *C. elegans* to PER and ETO are significantly closer than any of the two to IVM, confirmed by them being clustered together and having more enriched functions in common (Figure 6A; Table S10 in Text S1). The correlation of gene expression (across the 1,908 differentially expressed genes) between PER and ETO was 0.43, compared to 0.09 between PER and IVM (p<10^−10^ according to r-to-z Fisher test), showing that PER and ETO elicit a highly similar gene expression response to one another compared to the IVM treatment. PER and ETO cluster together since their targets (CPT-1 and CPT-2) act together to regulate fatty acid oxidation. The difference among PER and ETO, among others, was reflected by a small gene expression cluster near the top of the heatmap (Figure 6A), where we observed a group of genes downregulated in PER but upregulated in ETO. GO enrichment analysis on the genes related to this PER-specific downregulation pattern identified several enriched molecular functions (flavin-containing monooxygenase activity-GO:0004499; flavin adenine dinucleotide binding-GO:0050660; carbohydrate binding-GO:0030246 and NADP binding-GO:0050661) and biological processes (response to heat-GO:0009408; multicellular organismal development-GO:0007275). GO enrichment analysis was performed independently on the upregulated gene sets of each of the four treatments. The number of GO categories enriched in each treatment are shown in Figure 6B, and the specific GO terms in each intersection of Figure 6B can be found in Table S10 in Text S1. Two terms, one biological process (response to heat-GO:0009408) and one cellular component (peroxisome-GO:0005777) were enriched among genes upregulated in PER, ETO and PER+ETO, showing that both heat-responsive genes (primarily HSP70 genes) as well as genes related to peroxisome function were upregulated in all combinations of these treatments. Since CPT-1 is an initiating step in the translocation of long chain fatty acids across the mitochondrial membranes for beta-oxidation [44], [47] and the peroxisome proliferator activated receptor α (PPARα) is a nuclear receptor which stimulates genes involved in mitochondrial fatty acid oxidation and increases expression of those modulating pyruvate oxidation, the observed enrichment of genes related to peroxisome related activity is not surprising. Among the 10 GO terms which were only enriched among the PER+ETO treatment (but not in individual treatments) were two biological process terms related to fatty acid processes (fatty acid beta-oxidation-GO:0006635 and fatty acid metabolic process-GO:0006631), biological functions that are directly related to the function of CPT-1 and CPT-2.

### Docking of Perhexiline

**Figure 7:**
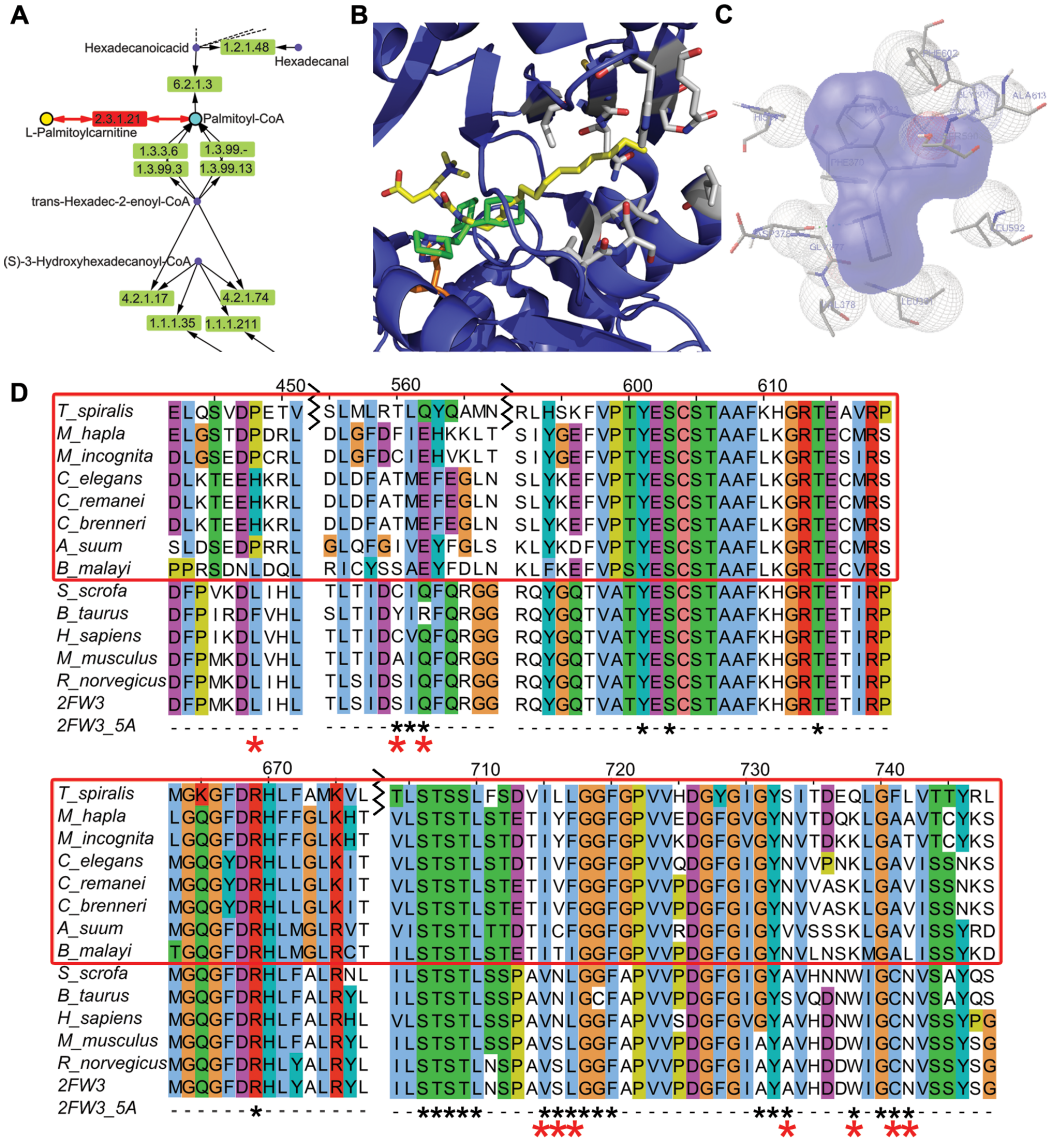
Docking of Perhexiline to rat carnitine palmitoyltransferase 2 and sequence alignment of carnitine palmitoyltransferase 2 from mammals and nematodes. **A.** Part of the fatty acid metabolic pathway in KEGG that includes the chokepoint reaction (chokepoint enzyme 2.3.1.21, with its substrate (L-Palmitoylcarnitine) and product (Palmitoyl-CoA)) shown in red. Perhexiline is believed to bind to 2.3.1.21. **B.** Docked structure of perhexiline (green) to CPT-2 (PDB ID: 2H4T), superimposed onto a CPT-2 structure with a bound drug, ST1326 (yellow) (PDB ID: 2FW3). Residues that differ between mammals and nematodes are shown in gray (L335, S445, Q447, V597, S598, L599, A615, W620, C623, N624), and the catalytic H372 is shown in orange. **C.** Interactions perhexiline make with CPT-2 (PDB ID: 2H4T) (P133, F134, M135, H372, D376, G377, V378, L381, S590, L592, G601). **D.** The *C. elegans* protein was used to find similar mammalian sequences with BLASTP and the non-redundant (NR) database. Residues shown in gray in B are labeled with red asterisks below the sequence. The alignment (using MUSCLE) of the following proteins is shown: gi|294805368|gb|ADF42518.1 (*S. scrofa*), gi|296489058|gb|DAA31171.1 (*B. Taurus*), gi|4503023|ref|NP_000089.1 (*H. sapiens*), gi|162138915|ref|NP_034079.2 (*M. musculus*), gi|1850592|gb|AAB48047.1 (*R. norvegicus*), 2FW3 chain A, Tsp_06820 (*T. spiralis*), Mh10g200708_Contig108_46414_50093 (*M. hapla*), prot_Minc00582 (*M. incognita*), R07H5.2a (*C. elegans*), gi|308491342|ref|XP_003107862.1 (*C. remanei*), gi|341894296|gb|EGT50231.1 (*C. brenneri*), gi|324506871|gb|ADY42921.1 (*A. suum*), 14424.m00388 (*B. malayi*).

The rat structure of CPT-2 (PDB ID: 2H4T) was used for the docking of Perhexiline, since that is the only species with crystal structures available. One major low-energy cluster with a binding energy of −5.8 kcal/mol resulted and contained 226 of the 250 genetic algorithm runs. Using Autodock 4 [41], Perhexiline was docked into the active site of CPT-2 [42] (Figure 7). The binding site of Perhexiline in CPT-2 does not overlap with the carnitine group in the ST-1326 (bound CPT-2 inhibitor in PDB ID: 2FW3) based on the docking calculations, but overlaps more with the fatty acid chain. The major contacts that Perhexiline makes in its docked configuration include: P133, F134, M135, F370, H372, D376, G377, V378, L381, S590, G601, and F602. H372 is the catalytic residue (Figure 7C). The amine group on Perhexiline makes a hydrogen bond with the backbone carbonyl group on D376. Residues that differ between mammals and nematode include L335, S445, Q447, V597, S598, L599, A615, W620, C623, N624 (Figure 7B).

## Discussion

Given the pressing need for new anthelmintic treatments and pesticides, this study outlines new potential drug targets of global importance found to be conserved in nematodes from different trophic ecologies as well as promising compounds that could lead to new anthelmintic treatments and nematicides. The targets offer the possibility for broad-spectrum drugs and pesticides for nematodes. We also provide a list of already known drugs that could be repositioned or further optimized as anthelmintics. Features of chokepoint enzymes that are known drug targets were analyzed. This is the first study to incorporate a large dataset of pan-phylum genomic data into a chokepoint analysis, provide a prioritized list of targets for broad-spectrum drugs, and test some of the prioritized drug-like compounds experimentally.

This work used the entire KEGG database to determine chokepoint reactions, then compared the homologous enzymes that are predicted to catalyze the chokepoint reactions in the intersection (CommNem) and union (UniNem) of the 10 nematode species with sequenced genomes, as well as drug targets in KEGG Drug and DrugBank. One caveat to this study is the possibility that the absence of complete pathway information may have led to false negative and false positive chokepoint drug targets. For instance, the entire deduced proteomes of some nematodes has not been mapped out due to the draft nature of the genome sequences (e.g. *B. malayi* genome used in this study). Some chokepoint reactions may utilize a chokepoint compound and produce a product that is also produced by several other reactions. To determine the effect of blocking the chokepoint reaction, modeling of the kinetics and equilibrium constants within the pathways would be required. However, these analyses are beyond the scope of this work. Another caveat surrounding the databases used in this study is the manner that compounds are linked to drug targets in KEGG Drug and DrugBank could yield false linkages between drugs and drug targets. For instance, DrugBank links drug targets and drugs using text-mining programs to search through abstracts in PubMed, as well as manual inspection by trained individuals. As the genomes and databases are improved, the analysis framework outlined here will become more powerful.

Despite limitations of the approach, two out of the six intestinal helminth drugs in the World Health Organization (WHO) Model list target enzymes that catalyze chokepoint reactions. The WHO Model List of Essential Medicines [48] contains a core list of minimum medicines that are needed for a basic health care system. The drugs in this list contain the most efficacious, safe, and cost-effective medicines for certain conditions. The presence of our predicted chokepoint drugs on this list indicates that chokepoint reactions may be useful in providing safe and effective treatments. The two drugs that target chokepoint reactions (listed with their respective targets) include: Levamisole (EC: 3.1.3.1 and EC: 6.1.1.6) and Praziquantel (EC: 2.5.1.18). The next two, Albendazole (DB00518) and Mebendazole target tubulin, which is not an enzyme. The remaining drugs, Niclosamide and Pyrantel, were not in DrugBank or in KEGG Drug and therefore, could not be identified in our study. In the category of antifilarials by the WHO, an additional 6 compounds are listed, but only two have EC associations. The two compounds, Suramin sodium (EC: 3.1.1.4 & 3.5.1.-) and Praziquantel (EC: 2.5.1.18), are both associated with targets that are enzymes involved in chokepoint reactions. Some of the enzyme drug targets are not in CommNem, but are in UniNem. Although it is not on the WHO list, Metrifonate is used as an insecticide and anthelmintic drug and targets an enzyme, EC: 3.1.1.7 (CommNem), which is associated with acetylcholinesterase in a chokepoint reaction.

Considering all anthelmintic drugs, there are also some drugs that are in KEGG that either do not have ECs associated or are not known chokepoints. Within KEGG Drug, Diethylcarbamazine (DB00711) targets two enzymes: EC: 1.9.3.1 (not in a chokepoint reaction) and EC: 1.13.11.34 (involved in a chokepoint reaction) [49]. Nitazoxanide targets EC: 1.2.1.51, which is not a known chokepoint enzyme [50]. Ivermectin (DB00602) and piperazine (DB00592) (two popular anthelminths) do not target enzymes, but target the GABA-A [51] and glutamate-gated chloride channels [52]. For Thiabendazole (DB00730), the metabolizing enzyme cytochrome P450 is a chokepoint. Thiabendazole is thought to inhibit fumarate reducatase [53] (EC: 1.3.99.1, which is not a chokepoint in our study), but the precise mode of action is unknown [54]. Biothionol, Oxamniquine, Niclosamide, Niridazole, and Triclabendazole are not found in DrugBank, and KEGG Drug does not have an EC number associated with them. If DrugBank is searched for drugs used to treat parasitic infections, eleven out of fifteen drugs used to treat parasitic infections that also have assigned ECs are chokepoint reactions in our study. An additional twelve drugs used to treat parasitic infections do not have ECs associated with them. Searching DrugBank for insecticides yielded four out of five drugs that have targets with ECs associated that are chokepoint reactions (Table S3 in Text S1).

Ideal drug targets in nematodes are proteins found only in nematodes and not in their host. The enzyme 4-coumarate-CoA ligase (4CL, EC: 6.2.1.12) is one such enzyme found in the CommNem group and not in *H. sapiens* in this study. This enzyme class has potential to be very interesting for pan-phylum nematicides. 4CL is involved in many reactions in the phenylpropanoid biosynthesis pathway, but the chokepoint compound is Cinnamoyl-CoA (C00540). Cinnamoyl-CoA feeds directly into the flavonoid biosynthetic pathway in plants and is also a precursor for capsaicin synthesis. The role 4CL plays in nematodes is unclear but it may be involved in host-parasite interactions (due to its position in the flavonoid pathway in plants) or in the production of antioxidants (due to its upstream involvement in capsaicin synthesis, to enable the worm to survive in the host).

During the course of this project, the chokepoint enzymes from the flatworm *Schistosoma mansoni* were published, and therefore our results were compared to theirs [55]. Out of 607 enzymes that were successfully placed in pathways, 120 were classified as chokepoint enzymes, and only 107 of these chokepoint enzymes were unique. From the chokepoint reactions found in KEGG in our study (2249), 56 chokepoint enzymes overlap with the *S. mansoni* chokepoint enzymes. Interestingly, there are many similarities between the nematode chokepoint reactions found in this study and the flatworms, with 50 and 33 chokepoint enzymes intersecting the *S. mansoni*/UniNem and CommNem sets (respectively). Only 1 chokepoint enzyme (EC:2.3.1.39) in the ParaNem set overlaps with the *S. mansoni* set, but it only obtained a target score of 1.

Several trends between chokepoint enzymes in general and chokepoint enzymes that have drugs associated with them were found. The chokepoint enzymes in CommNem and UniNem could potentially be enriched for drug targets by looking at trends in the KEGG Drug and DrugBank datasets. For instance, enzymes may be higher in priority because they were were significantly enriched in the set of chokepoint enzymes present in KEGG Drug and DrugBank, compared to AllKEGG and KEGGChoke. Ligases were significantly depleted relative to AllKEGG for both DrugBank and KEGG Drugs, so these enzymes would not be weighted as highly because they are depleted in databases of known drugs.

Whether the chokepoint compound was a substrate or a product of the chokepoint reaction did not seem to have any bearing on whether the enzyme was a good drug target. However, the pathway population was different between KEGGChoke compared to the DrugBank and KEGG Drug databases. Within the KEGG Drug and DrugBank databases, enzymes are involved in more pathways compared to KEGGChoke and AllKEGG. For KEGG Drug and DrugBank, enzymes involved in just one pathway are depleted and those involved in more than one are enriched for enzymes within the drug databases. A significant observation between the enzymes associated with chokepoint reactions in the drug databases and the entire list of chokepoint compounds (consumed and created) for various species is the position of the chokepoint in the pathway. Chokepoint enzymes that have known anthelmintic drugs associated with them are found more often around the first 20% (consumed compounds) or around 50% (created compounds) of the pathway length, and chokepoint enzymes that have compounds in KEGG drug associated with them were located around 70% of the pathway length. However, the trend did not exist for chokepoint enzymes associated with compounds in DrugBank, suggesting that this finding may have been an artifact of KEGG Drug. Before conclusions are drawn, the test should be expanded to other drug/protein databases.

Based on the areas studied (where significant differences were seen between a set of all chokepoint enzymes and the drug database), we developed a scoring scheme that helped us prioritize these chokepoint enzyme targets for experimental testing. Many of the targets can be considered broad spectrum, as these proteins are found in all 10 nematode genomes. For instance, nucleoside-triphosphate diphosphatase (EC: 3.6.1.19) scored high on the prioritized list. This enzyme is inhibited by plant compounds, lycorine and candimine, in *Trichomonas vaginalis*, a parasitic protozoan, which could make *T. vaginalis* more susceptible to the host immune system [56]. In addition, it is also a possible target for antimicrobial therapy [57]. In *S. mansoni*, EC: 3.6.1.19 is secreted and also believed to help the worm evade the immune system of the host; there is a new class of antischistosoma drugs (N-alkyl-aminoalkanethiosulfuric acids) that inhibit the enzyme and may negatively impact schistosoma survival [57]. Another prioritized target is nucleoside-diphosphate kinase (EC: 2.7.4.6), which is secreted by *T. spiralis* and may modulate host cell function [58]. This enzyme has been studied in *B. malayi* and is expressed during all parasitic stages in *B. malayi*, and molecular modeling for drug targeting has been performed for it in *B. malayi* [59].

Repositioning or further optimization of existing drugs may provide a means to obtain much needed anthelmintic drugs at a faster pace, as many of the drugs already have FDA approval. Existing drugs-like compounds may yield a faster path to anthelmintic drugs by providing a known scaffold that may require some optimization. Many drugs in KEGG Drug and DrugBank whose targets also hit nematode ECs have immunosuppresant, anti-inflammatory, antiviral, and antineoplastic activity. For example, Levamisole, an anthelmintic drug, is also a treatment for rheumatoid arthritis [60]. These target proteins may provide insight into how the parasite evades the immune system when it infects the host. Further, other targets with drugs that have immunosuppressant activity may yield a drug that has already been approved that can be repositioned as an anthelmintic drug. For instance, Mercaptopurine (DB01033) and Azathioprine (DB00993) (Table 1), which resulted from the prioritized list of drug-like compounds from DrugBank, both have immunosuppressive properties. In addition, several targets in KEGG Drug with homology to helminth proteins also have immunosuppressive activity, including IMP dehydrogenase (EC: 1.1.1.205). Several chokepoint targets from KEGG Drug with homology to helminth proteins also have antimalarial and antiprotozoal properties, such as phospholipase A2 (EC: 3.1.1.4). The corresponding drugs for various targets are listed in Table S6 in Text S1 and Table S7 in Text S1.

To find promising drug-like compounds for repositioning (or ones which hit scaffolds for which further optimization can be done), drug-like compounds that target chokepoint enzymes were also prioritized and the best candidates were tested in *C. elegans* and 2 parasitic nematodes. One compound, Perhexiline (PER) (DB01074) (**1**), yielded an EC50 value of 47.3 µM (18.5 ppm) and caused a slow movement and twitchy phenotype in *C. elegans*, as well as a deleterious phenotype in *H. contortus* and *O. lienalis*, two parasitic nematode species. PER is an approved small molecule drug which is used as a coronary vasodilator and used for angina treatment [19]. According to DrugBank, PER binds to *H. sapiens* carnitine o-palmitoyltransferase I (CPT-1) and carnitine o-palmitoyltransferase 2 (CPT-2). If PER inhibits CPT-1 or CPT-2 in living parasites, a drop in fatty acid oxidation can be measured by oxygen consumption rates experimentally. The dose-dependent decrease in basal oxygen consumption rates in the *C. elegans* exposed to PER (Figure 5A) provides indirect evidence that PER is acting via its intended mode of action on CPT-1 and CPT-2. In addition, a comparison of OCR in *C. elegans* exposed to either PER, ETO (or both) to an anthelmintic with a different mode of action would also provide an independent orthogonal confirmation of the similarity of PER and ETO in their possible mode of action on CPT-1 and CPT-2. Indeed, the lack of an observed decrease of OCR in *C. elegans* in the presence of IVM (which disrupts neurotransmission processes regulated by GluCl activity) further confirmed our hypothesis (Figure 5). Additionally, transcriptional responses to drugs often carry signatures for drug physiological mode of action. The transcriptional response to PER was measured by RNAseq and compared to that of ETO and IVM. Drugs with a related mechanism of action (i.e., PER and ETO) cluster together, since similar patterns of pathways are expected to be significantly perturbed. The clustering we observed (Figure 6A), as well as a Gene Ontology analysis of upregulated genes which (among other GO categories) includes peroxisome and fatty acid beta-oxidation, provides an additional confirmation of the similarity of PER and ETO in their mode of action (Table S10 in Text S1). Further experimentation, including in vitro enzyme assays, binding studies and drug resistance mutants, would need to be done to validate completely the mode of action and to move from hit to lead. The compound may need to be altered in order to increase efficacy.

There are 6 homologs of carnitine o-palmitoyltransferase (EC: 2.3.1.21) in *C. elegans*. R07H5.2 (*cpt-2*) is expressed in the adult and larval intestines of *C. elegans* and has an embryonic lethal RNAi phenotype, whereas Y46G5A.17 (*cpt-1*) does not have an RNAi phenotype (see below for a detailed explanation) and is expressed in the intestine, body wall muscle, and rectal gland cells in larva and in the pharynx, reproductive system, vulval muscle, and body wall muscle in adults [61]. The chokepoint compound in this reaction, L-palmitoylcarnitine (L-PC) has been shown to inhibit the Na/K pump in guinea-pig ventricular myocytes [62] and the interaction between L-PC and PIP2 in the membrane regulate K_ATP_ channels [63]. A module of the KEGG Fatty Acid Metabolism pathway map is shown in Figure 7A. Using Autodock 4 [41], perhexiline was docked into the active site of CPT-2 [42] (Figure 7B). The binding site of perhexiline in CPT-2 does not overlap with the carnitine group in the ST-1326 based on the docking calculations, which is consistant with biophysical experiments on CPT-1 [64], which showed competitive inhibition with respect to palmitoyl-CoA, but non-competitive inhibition with respect to carnitine. PER binds to residues in the active site that do not differ between mammals and nematodes (Figure 7D), which explains its efficacy in different phyla. Differences in residues between nematodes and mammals are present around the binding site (Figure 7D), and these differences could be exploited to generate a specific and more potent inhibitor by extending the PER molecule into this area.

**Figure 8:**
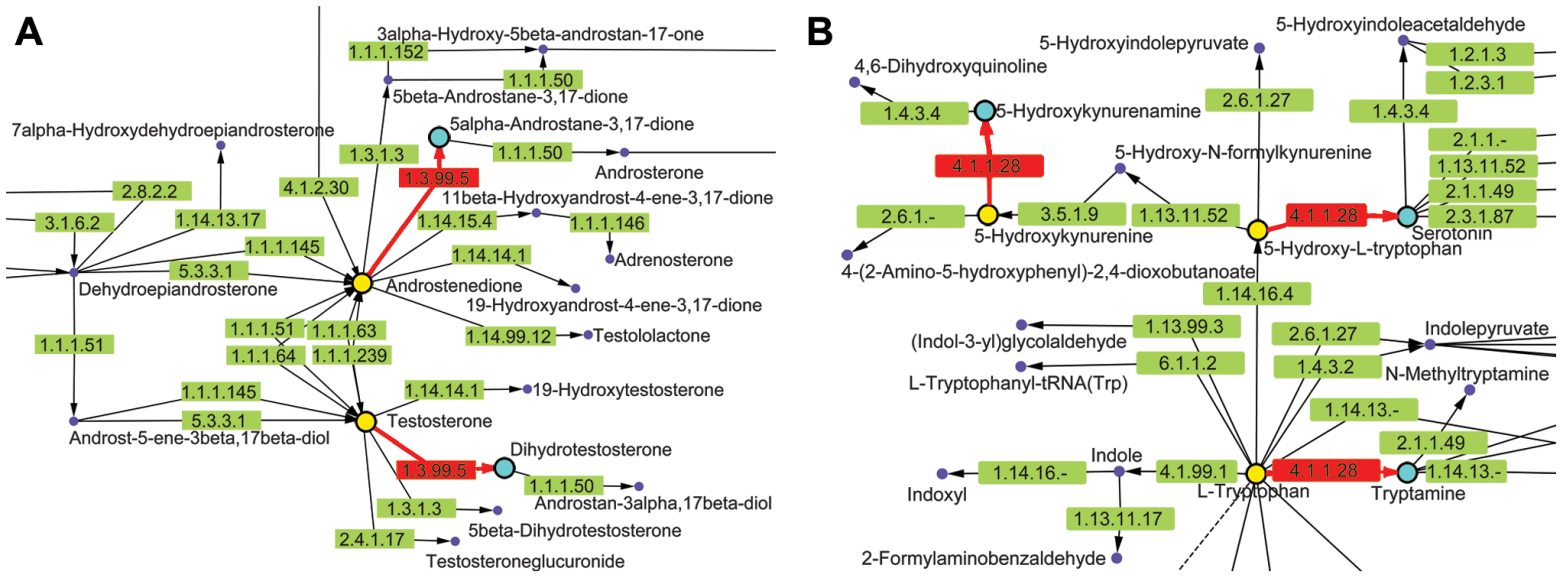
KEGG metabolic pathways containing chokepoint reactions for which a drug-like compound showed activity. Chokepoint reactions (chokepoint enzymes, with its substrate and product) are highlighted in red. **A.** Pathway Maps for Steroid Hormone Biosynthesis (ko00140). Chokepoint enzyme 1.3.99.5 is involved in a chokepoint reaction in this pathway. 5α-Androstane-3,17-dione (C00674) and 5α-Dihydro-testosterone (C03917) are chokepoint compounds. Azelic acid (drug-like compound **7**, DB00548) targets 3-oxo-5-alpha-steroid 4-dehydrogenase (EC 1.3.99.5) as well as other targets. Exposing *C. elegans* to azelic acid led to a movement-impaired phenotype. **B.** Pathway Maps for Tryptophan Metabolism (ko00380). Enzyme 4.1.1.28 is involved in a chokepoint reaction in this pathway. 5-Hydroxykynurenamine (C05638), Serotonin (C00780), and Trypamine (C00398) are chokepoint compounds. Carbidopa (drug-like compound **2**, DB00190) targets DOPA decarboxylase (EC 4.1.1.28). Exposing *C. elegans* to carbidopa produced a movement-impaired phenotype.

Two other compounds, Carbidopa (DB00190) (**2**) and Azelaic acid (DB00548) (**7**), also showed deleterious movement phenotypes in *C. elegans*. Carbidopa (**8**) is an approved small molecule that is an inhibitor of L-DOPA decarboxylase (EC: 4.1.1.28 chokepoint enzyme), which prevents the conversion of levodopa to dopamine (Figure 8). Carbidopa is used in the treatment of Parkinson's disease to reduce the side effects of levodopa, but has no anti-Parkinson actions by itself [19]. L-DOPA decarboxylase was also found to be a chokepoint in flatworm [55], and Methyldopa (**9**) has been found to inhibit enzyme activity in schistosoma extracts [65]. Methyldopa and carbidopa only differ by one amino group (Figure 3B). Azelaic acid is also an approved drug that targets, 3-oxo-5-alpha steroid 4 dehydrogenase (EC: 1.3.99.5 chokepoint enzyme), as well as thioredoxin reductase, tyrosinase, and DNA polymerase I [19]. Typically, azelaic acid is used to treat acne and has antimicrobial properties.

High throughput RNAi studies in *C. elegans* can provide evidence that an enzyme has an important *in vivo* function [66], suggesting that targeting that enzyme using a drug would likely have a deleterious effect on the worm. Similar phenotypes observed for a drug treatment and RNAi (or gene mutation) provide support that the drug is specifically inhibiting the gene product targeted by RNAi. However, high-throughput RNAi data needs to be considered with caution [66], [67], [68], and thus it was not possible to incorporate it into our chemogenomic pipeline. There are a number of reasons why high throughput RNAi experiments can fail to generate a phenotype. One biological reason is that there is a family of genes that encode the enzymatic activity, and knockdown of any single gene will have no effect due to genetic redundancy. This appears to the case for L-DOPA decarboxylase (EC: 4.1.1.28), which is encoded by three paralogs (K01C8.3, ZK829.2, and C05D2.3) that do not show single gene RNAi phenotypes. In some cases however, gene family members can have essential functions, due to divergent protein sequences, subcellular compartmentalized functions and/or unique expression behavior, which may explain why *cpt-2* RNAi displays a strong phenotype. Other biological reasons why RNAi may fail to display a phenotype include RNAi resistance for the genes or that the relevant functional cell type is largely resist to RNAi (e.g. neurons). Additionally, high throughput RNAi in *C. elegans* has, methodologically, a relatively high rate of false negatives. In contrast, the false positive rate for RNAi in *C. elegans* is generally low, but can occur due to the libraries containing some incorrect RNAi clone IDs. Thus while high-throughput RNAi data can be used as a starting point, gene product hits from chemogenomic pipelines must be individually tested experimentally, including verification of RNAi clone identity, assessment of the extent of knockdown, or through analysis of gene deletions, if available. Finally, when comparing phenotypes generated by RNAi (or mutant) relative to drug treatment, the extent of gene product loss of function and drug-mediated inhibition need to be comparable, with consideration of the developmental stage that is being examined. For the compounds with hits, further experimentation that includes other life cycle stages would need to be performed to determine if the compounds should progress to advanced testing and move it from ‘hit’ to ‘lead’. The compound may also need to be modified to increase efficacy.

This study has yielded many interesting lead drug target hits and drug-like compounds that should be explored further that could potentially yield a next-generation anthelmintic/nematicide or novel drug target.

### Conclusions

In this study, we report chokepoint reactions and enzymes that are common to all 10 studied species of nematodes, as well as chokepoint reactions and enzymes that encompass the union of the 10 nematode species. This study goes further than previous studies to try to understand features of chokepoint enzymes that are successful drugs targets, then uses available diverse information to prioritize the nematode chokepoint enzymes for those that are good drug candidates. Scoring high on the prioritized list are targets that are under investigation for treatment of parasites, indicating that the list contains reasonable targets that should be investigated further. In addition, KEGG Drug and DrugBank were examined for existing drugs that could be repositioned or optimized as anthelmintic drugs. Three of the seven compounds were experimentally tested and show efficacy in *C. elegans*, and one of these three (Perhexiline) shows efficacy in two nematode species with distinct modes of parasitism. A suggested mode of action was also outlined for Perhexiline. Computational modeling results suggest opportunities for higher affinity and specificity using this compound as a starting point. The list of prioritized drug targets and drug compounds has enormous potential for the development of new and urgently-needed anthelmintic drugs and pesticides.

## Supporting Information

Text S1 **Supporting tables.** Table S1. ECs of the 169 chokepoints from the intersection of the nematode deduced proteomes. Table S2. ECs of the 477 chokepoints identified in the union of the nematode deduced proteomes. Table S3. Insecticides in DrugBank. Chokepoint drugs are listed in bold. Table S4. Antiparasitic Drugs in DrugBank^a^. Table S5: KEGG drugs targets that hit nematode enzymes. The corresponding drugs are listed in Table S3 and Table S4. Table S6. KEGG drugs that hit Common Nematodes ECs. Table S7. Additional KEGG drugs that hit from UniNem. Table S8. Top prioritized enzymes (ParaNem). Table S9. RNAseq reads stats and differential expressed genes. Table S10. Gene Ontology enriched categories of upregulated *C. elegans* genes in presence of PER, ETO, PER+ETO and IVM.
(DOCX)

Text S2 **Supporting figures.** Figure S1. Chokepoints found in various groups. The total number of ECs that were mapped from AllKEGG and the number of those that are in KEGGChoke are shown in the two lighter grey colors. The number of chokepoint targets in the various groups that have ECs associated with a drug in KEGG Drug (dark grey) and DrugBank (black). CommNem, intersection of nematode ECs; UniNem, set of all nematode ECs; Hs, ECs from *H. sapiens*; Dm, ECs from *D. melanogaster*. Figure S2. Enriched and depleted enzyme categories based on EC nomenclature in various groups and species. Heatmap illustrating enrichment or depletion in the groups for A. chokepoint enzymes within KEGG (KEGGChoke) and B. enzymes from all of KEGG (AllKEGG). The extreme blue color indicates that the enzyme category was significantly depleted and the extreme red color indicates the enzyme category was significantly enriched relative to either KEGGChoke or AllKEGG using Fisher's Exact Test. The intermediate color shades indicate enrichment or depletion, but are not statistically significant. CommNem, intersection of ECs; UniNem, set of all nematode ECs; Hs, ECs from *H. sapiens*; Dm, ECs from *D. melanogaster*; DrugBank, ECs from DrugBank; KEGG Drug, ECs from KEGG Drug. Figure S3. Number of pathways in which an enzyme acts. The data is broken into enzymes acting in one pathway (light grey) versus multiple pathways (dark grey). Comparison of percentage of ECs involved in one versus multiple pathways in A. UniNem, CommNem, KEGGChoke, and AllKEGG and B. KEGG Drug, DrugBank, KEGGChoke, and AllKEGG. CommNem, intersection of ECs; UniNem, set of all nematode ECs; KEGGChoke, chokepoint enzymes within KEGG; AllKEGG, all enzymes within KEGG; DrugBank, ECs from DrugBank; KEGG Drug, ECs from KEGG Drug.
(DOCX)

Video S1 ***C. elegans*** **control.**
(M4V)

Video S2 ***C. elegans*** **exposed to compound 1.**
(M4V)

Video S3 ***C. elegans*** **exposed to compound 2.**
(M4V)

Video S4 ***C. elegans*** **exposed to compound 7.**
(M4V)

Video S5 ***H. controtus*** **control.**
(M4V)

Video S6 ***H. controtus*** **exposed to compound 1.**
(M4V)

Video S7 ***H. controtus*** **exposed to compound 2.**
(M4V)

Video S8 ***H. controtus*** **exposed to compound 7.**
(M4V)
