# Supplementary material for: Discovery of Anthelmintic Drug Targets and Drugs Using Chokepoints in Nematode Metabolic Pathways
Source: PLoS Pathog. 2013 Aug 1;9(8):e1003505. doi: 10.1371/journal.ppat.1003505 (PMC3731235; doi:10.1371/journal.ppat.1003505)
Supplement: Text S1 — Supporting tables. Table S1. ECs of the 169 chokepoints from the intersection of the nematode deduced proteomes. Table S2. ECs of the 477 chokepoints identified in the union of the nematode deduced proteomes. Table S3. Insecticides in DrugBank. Chokepoint drugs are listed in bold. Table S4. Antiparasitic Drugs in DrugBanka. Table S5: KEGG drugs targets that hit nematode enzymes. The corresponding drugs are listed in Table S3 and Table S4. Table S6. KEGG drugs that hit Common Nematodes ECs. Table S7. Additional KEGG drugs that hit from UniNem. Table S8. Top prioritized enzymes (ParaNem). Table S9. RNAseq reads stats and differential expressed genes. Table S10. Gene Ontology enriched categories of upregulated C. elegans genes in presence of PER, ETO, PER+ETO and IVM. (DOCX) [file ppat.1003505.s001.docx]

**SUPPORTING INFORATION**

**Supporting Tables:**

**Table S1. ECs of the 169 chokepoints from the intersection of the nematode deduced proteomes.**

| 1.1.-.- |  | 2.1.1.- |  | 2.7.1.15 |  | 3.1.4.- |  | 4.6.1.1 |
| --- | --- | --- | --- | --- | --- | --- | --- | --- |
| 1.1.1.- |  | 2.1.1.37 |  | 2.7.1.40 |  | 3.1.4.17 |  | 4.99.1.1 |
| 1.1.1.1 |  | 2.1.1.43 |  | 2.7.1.48 |  | 3.2.1.- |  | 5.3.1.23 |
| 1.1.1.100 |  | 2.1.2.10 |  | 2.7.3.3 |  | 3.2.1.14 |  | 5.3.2.1 |
| 1.1.1.153 |  | 2.2.1.1 |  | 2.7.4.6 |  | 3.2.1.3 |  | 5.3.3.8 |
| 1.1.1.205 |  | 2.3.1.- |  | 2.7.6.3 |  | 3.2.1.31 |  | 5.4.2.1 |
| 1.1.1.271 |  | 2.3.1.12 |  | 2.7.7.- |  | 3.2.1.52 |  | 6.1.1.1 |
| 1.1.1.35 |  | 2.3.1.16 |  | 2.7.7.1 |  | 3.3.2.1 |  | 6.1.1.10 |
| 1.1.1.42 |  | 2.3.1.168 |  | 2.7.7.15 |  | 3.3.2.9 |  | 6.1.1.11 |
| 1.1.1.95 |  | 2.3.1.181 |  | 2.7.7.23 |  | 3.4.-.- |  | 6.1.1.12 |
| 1.11.1.7 |  | 2.3.1.21 |  | 2.7.7.4 |  | 3.4.11.1 |  | 6.1.1.14 |
| 1.11.1.9 |  | 2.3.1.24 |  | 2.7.7.41 |  | 3.5.1.- |  | 6.1.1.15 |
| 1.14.-.- |  | 2.3.1.43 |  | 2.7.8.- |  | 3.5.1.4 |  | 6.1.1.17 |
| 1.14.11.- |  | 2.3.1.50 |  | 2.7.8.1 |  | 3.5.2.2 |  | 6.1.1.19 |
| 1.14.11.4 |  | 2.3.1.51 |  | 2.7.8.5 |  | 3.5.4.5 |  | 6.1.1.2 |
| 1.14.13.- |  | 2.3.1.61 |  | 2.8.1.7 |  | 3.5.5.1 |  | 6.1.1.20 |
| 1.14.13.8 |  | 2.3.1.76 |  | 2.8.2.- |  | 3.6.1.- |  | 6.1.1.21 |
| 1.14.14.1 |  | 2.3.1.9 |  | 2.8.4.1 |  | 3.6.1.14 |  | 6.1.1.22 |
| 1.14.17.1 |  | 2.3.2.2 |  | 3.1.1.- |  | 3.6.1.17 |  | 6.1.1.3 |
| 1.14.18.1 |  | 2.4.1.- |  | 3.1.1.1 |  | 3.6.1.19 |  | 6.1.1.4 |
| 1.2.1.31 |  | 2.4.1.17 |  | 3.1.1.17 |  | 3.6.1.28 |  | 6.1.1.5 |
| 1.2.1.5 |  | 2.4.2.- |  | 3.1.1.31 |  | 3.6.1.29 |  | 6.1.1.6 |
| 1.2.4.1 |  | 2.4.2.10 |  | 3.1.1.4 |  | 4.1.1.28 |  | 6.1.1.7 |
| 1.2.4.2 |  | 2.4.2.3 |  | 3.1.1.47 |  | 4.1.1.36 |  | 6.1.1.9 |
| 1.2.4.4 |  | 2.4.2.8 |  | 3.1.1.7 |  | 4.1.2.13 |  | 6.2.1.- |
| 1.3.1.- |  | 2.5.1.- |  | 3.1.2.- |  | 4.1.2.27 |  | 6.2.1.1 |
| 1.3.1.38 |  | 2.5.1.1 |  | 3.1.3.- |  | 4.2.1.1 |  | 6.2.1.12 |
| 1.3.3.6 |  | 2.5.1.18 |  | 3.1.3.11 |  | 4.2.1.17 |  | 6.3.2.- |
| 1.3.99.- |  | 2.5.1.2 |  | 3.1.3.18 |  | 4.2.1.3 |  | 6.3.2.2 |
| 1.3.99.3 |  | 2.6.1.1 |  | 3.1.3.25 |  | 4.2.1.46 |  | 6.3.4.4 |
| 1.4.4.2 |  | 2.6.1.7 |  | 3.1.3.4 |  | 4.2.1.70 |  | 6.3.5.6 |
| 1.7.2.1 |  | 2.7.1.- |  | 3.1.3.5 |  | 4.2.1.9 |  | 6.3.5.7 |
| 1.8.1.4 |  | 2.7.1.108 |  | 3.1.3.56 |  | 4.3.2.2 |  | 6.4.1.2 |
| 1.8.4.2 |  | 2.7.1.149 |  | 3.1.3.67 |  | 4.4.1.17 |  |  |

**Table S2. ECs of the 477 chokepoints identified in the union of the nematode deduced proteomes.**

| EC | C. brenneri | C. briggsae | C. elegans | C. japonicum | C. remanei | P. pacificus | M. hapla | M. incognita | B. malayi | T. spiralis | # of Species |
| --- | --- | --- | --- | --- | --- | --- | --- | --- | --- | --- | --- |
| 1.1.-.- | Y | Y | Y | Y | Y | Y | Y | Y | Y | Y | 10 |
| 1.1.1.- | Y | Y | Y | Y | Y | Y | Y | Y | Y | Y | 10 |
| 1.1.1.1 | Y | Y | Y | Y | Y | Y | Y | Y | Y | Y | 10 |
| 1.1.1.100 | Y | Y | Y | Y | Y | Y | Y | Y | Y | Y | 10 |
| 1.1.1.102 | Y | Y | - | Y | Y | Y | - | Y | Y | Y | 8 |
| 1.1.1.103 | - | Y | Y | Y | Y | Y | - | - | Y | Y | 7 |
| 1.1.1.122 | Y | Y | Y | Y | Y | Y | - | - | - | - | 6 |
| 1.1.1.125 | Y | Y | Y | Y | Y | Y | Y | Y | Y | - | 9 |
| 1.1.1.145 | Y | Y | Y | Y | Y | - | - | - | - | Y | 6 |
| 1.1.1.153 | Y | Y | Y | Y | Y | Y | Y | Y | Y | Y | 10 |
| 1.1.1.170 | - | - | - | Y | - | - | - | - | - | - | 1 |
| 1.1.1.193 | - | Y | - | Y | - | - | - | Y | - | - | 3 |
| 1.1.1.205 | Y | Y | Y | Y | Y | Y | Y | Y | Y | Y | 10 |
| 1.1.1.206 | Y | - | - | - | - | - | - | - | - | - | 1 |
| 1.1.1.21 | Y | Y | Y | Y | Y | Y | Y | Y | Y | - | 9 |
| 1.1.1.219 | Y | Y | Y | Y | Y | Y | Y | Y | Y | - | 9 |
| 1.1.1.27 | Y | Y | Y | Y | Y | - | Y | - | Y | Y | 8 |
| 1.1.1.271 | Y | Y | Y | Y | Y | Y | Y | Y | Y | Y | 10 |
| 1.1.1.30 | - | - | - | - | - | - | - | - | Y | - | 1 |
| 1.1.1.31 | Y | Y | Y | Y | Y | Y | Y | Y | - | - | 8 |
| 1.1.1.35 | Y | Y | Y | Y | Y | Y | Y | Y | Y | Y | 10 |
| 1.1.1.4 | - | - | - | - | Y | - | - | - | - | - | 1 |
| 1.1.1.42 | Y | Y | Y | Y | Y | Y | Y | Y | Y | Y | 10 |
| 1.1.1.57 | - | - | - | Y | - | - | - | - | - | - | 1 |
| 1.1.1.58 | - | - | - | Y | - | - | - | - | - | - | 1 |
| 1.1.1.85 | - | - | - | Y | - | - | - | - | - | - | 1 |
| 1.1.1.95 | Y | Y | Y | Y | Y | Y | Y | Y | Y | Y | 10 |
| 1.1.3.8 | - | - | - | Y | - | - | - | - | - | - | 1 |
| 1.1.99.- | - | - | - | Y | Y | Y | - | - | - | - | 3 |
| 1.10.3.- | - | - | - | Y | Y | - | - | - | - | - | 2 |
| 1.11.1.12 | - | - | - | - | - | - | - | - | - | Y | 1 |
| 1.11.1.6 | Y | Y | Y | Y | Y | Y | Y | Y | - | Y | 9 |
| 1.11.1.7 | Y | Y | Y | Y | Y | Y | Y | Y | Y | Y | 10 |
| 1.11.1.8 | - | - | - | - | - | - | Y | - | - | - | 1 |
| 1.11.1.9 | Y | Y | Y | Y | Y | Y | Y | Y | Y | Y | 10 |
| 1.13.-.- | - | Y | - | - | Y | - | - | - | - | - | 2 |
| 1.13.11.- | - | - | - | Y | Y | Y | - | - | - | - | 3 |
| 1.13.11.11 | Y | Y | Y | Y | Y | Y | Y | Y | - | - | 8 |
| 1.13.11.19 | - | - | - | - | - | - | Y | - | - | Y | 2 |
| 1.13.11.20 | Y | Y | Y | Y | Y | Y | Y | Y | - | - | 8 |
| 1.13.11.39 | - | - | - | Y | - | - | - | - | - | - | 1 |
| 1.13.11.5 | Y | Y | Y | Y | Y | Y | Y | Y | - | - | 8 |
| 1.13.11.52 | - | - | - | - | - | Y | - | - | Y | Y | 3 |
| 1.13.11.53 | Y | - | Y | - | - | - | - | - | - | - | 2 |
| 1.13.11.6 | Y | Y | Y | Y | Y | Y | - | - | - | - | 6 |
| 1.14.-.- | Y | Y | Y | Y | Y | Y | Y | Y | Y | Y | 10 |
| 1.14.11.- | Y | Y | Y | Y | Y | Y | Y | Y | Y | Y | 10 |
| 1.14.11.1 | Y | Y | Y | Y | Y | Y | Y | Y | - | - | 8 |
| 1.14.11.13 | - | - | - | - | - | Y | - | - | - | - | 1 |
| 1.14.11.4 | Y | Y | Y | Y | Y | Y | Y | Y | Y | Y | 10 |
| 1.14.12.10 | - | - | - | Y | - | - | - | - | - | - | 1 |
| 1.14.13.- | Y | Y | Y | Y | Y | Y | Y | Y | Y | Y | 10 |
| 1.14.13.13 | - | Y | - | Y | - | - | - | - | - | - | 2 |
| 1.14.13.39 | Y | Y | Y | Y | Y | - | Y | - | Y | Y | 8 |
| 1.14.13.50 | - | - | - | Y | - | - | - | - | - | - | 1 |
| 1.14.13.72 | Y | Y | Y | Y | Y | Y | - | - | - | - | 6 |
| 1.14.13.8 | Y | Y | Y | Y | Y | Y | Y | Y | Y | Y | 10 |
| 1.14.13.83 | - | - | - | Y | - | - | - | - | - | - | 1 |
| 1.14.13.9 | Y | Y | Y | Y | Y | Y | - | - | - | - | 6 |
| 1.14.14.1 | Y | Y | Y | Y | Y | Y | Y | Y | Y | Y | 10 |
| 1.14.15.- | - | Y | Y | Y | Y | Y | Y | Y | Y | Y | 9 |
| 1.14.16.2 | Y | Y | Y | Y | Y | Y | Y | - | Y | Y | 9 |
| 1.14.16.4 | Y | Y | Y | Y | Y | Y | Y | - | - | Y | 8 |
| 1.14.17.1 | Y | Y | Y | Y | Y | Y | Y | Y | Y | Y | 10 |
| 1.14.18.1 | Y | Y | Y | Y | Y | Y | Y | Y | Y | Y | 10 |
| 1.14.19.- | Y | Y | Y | Y | Y | Y | Y | Y | Y | - | 9 |
| 1.14.19.1 | Y | Y | Y | Y | Y | Y | Y | Y | Y | - | 9 |
| 1.14.19.3 | - | - | - | - | - | Y | - | - | - | - | 1 |
| 1.14.21.6 | - | - | - | Y | - | - | - | - | - | - | 1 |
| 1.14.99.- | Y | Y | Y | Y | Y | Y | - | Y | - | - | 7 |
| 1.14.99.3 | - | - | - | Y | - | - | - | - | - | - | 1 |
| 1.14.99.38 | Y | Y | Y | Y | Y | Y | - | - | - | - | 6 |
| 1.16.8.1 | Y | Y | Y | Y | Y | - | - | - | Y | - | 6 |
| 1.2.-.- | - | - | - | Y | - | - | - | - | - | - | 1 |
| 1.2.1.- | Y | Y | Y | Y | Y | Y | Y | Y | Y | - | 9 |
| 1.2.1.3 | Y | Y | Y | Y | Y | Y | Y | Y | - | Y | 9 |
| 1.2.1.31 | Y | Y | Y | Y | Y | Y | Y | Y | Y | Y | 10 |
| 1.2.1.41 | Y | Y | Y | Y | Y | Y | Y | Y | - | Y | 9 |
| 1.2.1.5 | Y | Y | Y | Y | Y | Y | Y | Y | Y | Y | 10 |
| 1.2.3.1 | - | - | - | - | - | Y | - | - | - | - | 1 |
| 1.2.4.1 | Y | Y | Y | Y | Y | Y | Y | Y | Y | Y | 10 |
| 1.2.4.2 | Y | Y | Y | Y | Y | Y | Y | Y | Y | Y | 10 |
| 1.2.4.4 | Y | Y | Y | Y | Y | Y | Y | Y | Y | Y | 10 |
| 1.3.-.- | Y | Y | Y | Y | Y | Y | Y | - | Y | - | 8 |
| 1.3.1.- | Y | Y | Y | Y | Y | Y | Y | Y | Y | Y | 10 |
| 1.3.1.2 | Y | Y | Y | Y | Y | Y | Y | Y | - | - | 8 |
| 1.3.1.28 | Y | - | - | - | - | - | - | - | - | - | 1 |
| 1.3.1.33 | - | - | - | - | Y | Y | - | - | - | - | 2 |
| 1.3.1.38 | Y | Y | Y | Y | Y | Y | Y | Y | Y | Y | 10 |
| 1.3.1.70 | Y | Y | Y | Y | Y | Y | - | - | - | - | 6 |
| 1.3.1.72 | Y | Y | Y | Y | Y | Y | Y | Y | - | - | 8 |
| 1.3.1.76 | - | - | - | Y | - | - | - | - | - | - | 1 |
| 1.3.3.4 | Y | - | Y | Y | Y | Y | - | - | - | - | 5 |
| 1.3.3.6 | Y | Y | Y | Y | Y | Y | Y | Y | Y | Y | 10 |
| 1.3.5.2 | Y | Y | Y | Y | Y | Y | Y | - | Y | - | 8 |
| 1.3.99.- | Y | Y | Y | Y | Y | Y | Y | Y | Y | Y | 10 |
| 1.3.99.23 | - | - | - | - | - | - | - | - | Y | - | 1 |
| 1.3.99.3 | Y | Y | Y | Y | Y | Y | Y | Y | Y | Y | 10 |
| 1.3.99.5 | Y | Y | Y | Y | Y | Y | Y | Y | Y | - | 9 |
| 1.4.1.- | Y | Y | Y | Y | Y | - | - | - | Y | - | 6 |
| 1.4.1.9 | - | - | - | Y | - | - | - | - | - | - | 1 |
| 1.4.3.- | - | - | - | Y | - | - | - | - | - | - | 1 |
| 1.4.3.1 | Y | Y | Y | Y | - | Y | - | - | - | - | 5 |
| 1.4.3.3 | Y | Y | Y | Y | Y | Y | Y | Y | - | - | 8 |
| 1.4.3.4 | Y | Y | Y | Y | Y | Y | - | - | - | - | 6 |
| 1.4.4.2 | Y | Y | Y | Y | Y | Y | Y | Y | Y | Y | 10 |
| 1.5.99.2 | Y | Y | Y | Y | Y | Y | Y | - | Y | - | 8 |
| 1.7.2.1 | Y | Y | Y | Y | Y | Y | Y | Y | Y | Y | 10 |
| 1.8.1.4 | Y | Y | Y | Y | Y | Y | Y | Y | Y | Y | 10 |
| 1.8.1.9 | Y | Y | Y | Y | Y | Y | - | - | Y | Y | 8 |
| 1.8.4.1 | Y | Y | Y | - | Y | Y | - | Y | Y | - | 7 |
| 1.8.4.2 | Y | Y | Y | Y | Y | Y | Y | Y | Y | Y | 10 |
| 2.1.1.- | Y | Y | Y | Y | Y | Y | Y | Y | Y | Y | 10 |
| 2.1.1.1 | Y | Y | Y | Y | Y | Y | Y | - | Y | - | 8 |
| 2.1.1.103 | Y | Y | Y | Y | Y | Y | Y | - | - | - | 7 |
| 2.1.1.107 | - | - | - | Y | - | - | - | - | - | - | 1 |
| 2.1.1.114 | Y | Y | Y | Y | Y | Y | Y | - | Y | Y | 9 |
| 2.1.1.13 | Y | Y | Y | Y | Y | Y | - | - | - | Y | 7 |
| 2.1.1.130 | - | - | - | Y | - | - | - | - | - | - | 1 |
| 2.1.1.131 | - | - | - | Y | - | - | - | - | - | - | 1 |
| 2.1.1.132 | - | - | - | Y | - | - | - | - | - | - | 1 |
| 2.1.1.133 | - | - | - | Y | - | - | - | - | - | - | 1 |
| 2.1.1.143 | - | - | Y | - | - | Y | - | Y | - | - | 3 |
| 2.1.1.37 | Y | Y | Y | Y | Y | Y | Y | Y | Y | Y | 10 |
| 2.1.1.41 | Y | Y | - | Y | Y | - | - | - | Y | - | 5 |
| 2.1.1.43 | Y | Y | Y | Y | Y | Y | Y | Y | Y | Y | 10 |
| 2.1.1.49 | - | Y | Y | Y | Y | - | Y | Y | - | - | 6 |
| 2.1.1.64 | - | - | - | Y | - | - | - | - | - | - | 1 |
| 2.1.2.10 | Y | Y | Y | Y | Y | Y | Y | Y | Y | Y | 10 |
| 2.2.1.1 | Y | Y | Y | Y | Y | Y | Y | Y | Y | Y | 10 |
| 2.3.-.- | Y | Y | Y | Y | Y | Y | - | Y | - | - | 7 |
| 2.3.1.- | Y | Y | Y | Y | Y | Y | Y | Y | Y | Y | 10 |
| 2.3.1.102 | - | - | - | Y | - | - | - | - | - | - | 1 |
| 2.3.1.12 | Y | Y | Y | Y | Y | Y | Y | Y | Y | Y | 10 |
| 2.3.1.135 | - | - | - | - | - | Y | - | Y | Y | - | 3 |
| 2.3.1.16 | Y | Y | Y | Y | Y | Y | Y | Y | Y | Y | 10 |
| 2.3.1.168 | Y | Y | Y | Y | Y | Y | Y | Y | Y | Y | 10 |
| 2.3.1.176 | Y | Y | Y | Y | Y | Y | Y | Y | Y | - | 9 |
| 2.3.1.181 | Y | Y | Y | Y | Y | Y | Y | Y | Y | Y | 10 |
| 2.3.1.21 | Y | Y | Y | Y | Y | Y | Y | Y | Y | Y | 10 |
| 2.3.1.22 | Y | Y | Y | Y | Y | Y | - | Y | Y | Y | 9 |
| 2.3.1.24 | Y | Y | Y | Y | Y | Y | Y | Y | Y | Y | 10 |
| 2.3.1.26 | Y | Y | Y | Y | Y | Y | - | Y | - | Y | 8 |
| 2.3.1.29 | Y | Y | Y | Y | Y | Y | - | - | Y | Y | 8 |
| 2.3.1.30 | - | - | - | Y | Y | - | - | - | - | - | 2 |
| 2.3.1.39 | - | - | - | - | - | - | Y | - | - | Y | 2 |
| 2.3.1.41 | - | - | - | Y | Y | - | - | - | - | - | 2 |
| 2.3.1.42 | Y | Y | Y | - | Y | Y | Y | Y | Y | - | 8 |
| 2.3.1.43 | Y | Y | Y | Y | Y | Y | Y | Y | Y | Y | 10 |
| 2.3.1.47 | - | - | - | Y | Y | - | - | - | - | - | 2 |
| 2.3.1.50 | Y | Y | Y | Y | Y | Y | Y | Y | Y | Y | 10 |
| 2.3.1.51 | Y | Y | Y | Y | Y | Y | Y | Y | Y | Y | 10 |
| 2.3.1.6 | Y | Y | Y | Y | Y | Y | Y | Y | Y | - | 9 |
| 2.3.1.61 | Y | Y | Y | Y | Y | Y | Y | Y | Y | Y | 10 |
| 2.3.1.65 | Y | Y | Y | - | Y | - | - | - | - | - | 4 |
| 2.3.1.76 | Y | Y | Y | Y | Y | Y | Y | Y | Y | Y | 10 |
| 2.3.1.85 | Y | Y | Y | Y | Y | Y | Y | - | Y | Y | 9 |
| 2.3.1.9 | Y | Y | Y | Y | Y | Y | Y | Y | Y | Y | 10 |
| 2.3.1.94 | Y | Y | Y | Y | Y | Y | - | Y | Y | - | 8 |
| 2.3.2.2 | Y | Y | Y | Y | Y | Y | Y | Y | Y | Y | 10 |
| 2.3.2.4 | Y | Y | Y | - | Y | Y | Y | - | - | - | 6 |
| 2.4.1.- | Y | Y | Y | Y | Y | Y | Y | Y | Y | Y | 10 |
| 2.4.1.117 | Y | - | Y | Y | Y | Y | Y | Y | Y | Y | 9 |
| 2.4.1.129 | - | - | Y | Y | Y | Y | - | - | - | - | 4 |
| 2.4.1.17 | Y | Y | Y | Y | Y | Y | Y | Y | Y | Y | 10 |
| 2.4.1.22 | - | - | - | Y | - | - | Y | Y | Y | Y | 5 |
| 2.4.1.227 | - | - | - | Y | - | - | - | - | - | - | 1 |
| 2.4.1.5 | - | Y | - | - | - | Y | - | - | Y | - | 3 |
| 2.4.1.83 | Y | Y | Y | Y | Y | Y | Y | Y | Y | - | 9 |
| 2.4.2.- | Y | Y | Y | Y | Y | Y | Y | Y | Y | Y | 10 |
| 2.4.2.10 | Y | Y | Y | Y | Y | Y | Y | Y | Y | Y | 10 |
| 2.4.2.18 | - | - | - | Y | - | - | - | - | - | - | 1 |
| 2.4.2.21 | Y | Y | Y | Y | Y | Y | Y | Y | - | - | 8 |
| 2.4.2.3 | Y | Y | Y | Y | Y | Y | Y | Y | Y | Y | 10 |
| 2.4.2.7 | Y | Y | Y | Y | Y | Y | Y | Y | Y | - | 9 |
| 2.4.2.8 | Y | Y | Y | Y | Y | Y | Y | Y | Y | Y | 10 |
| 2.5.1.- | Y | Y | Y | Y | Y | Y | Y | Y | Y | Y | 10 |
| 2.5.1.1 | Y | Y | Y | Y | Y | Y | Y | Y | Y | Y | 10 |
| 2.5.1.15 | - | - | - | Y | - | - | - | - | - | - | 1 |
| 2.5.1.16 | Y | Y | Y | Y | Y | Y | Y | Y | Y | - | 9 |
| 2.5.1.17 | Y | Y | Y | Y | Y | Y | - | - | - | Y | 7 |
| 2.5.1.18 | Y | Y | Y | Y | Y | Y | Y | Y | Y | Y | 10 |
| 2.5.1.2 | Y | Y | Y | Y | Y | Y | Y | Y | Y | Y | 10 |
| 2.5.1.26 | Y | Y | Y | Y | Y | Y | Y | Y | Y | - | 9 |
| 2.5.1.29 | - | - | - | - | - | Y | - | - | Y | Y | 3 |
| 2.5.1.3 | - | - | - | Y | Y | - | - | - | - | - | 2 |
| 2.5.1.30 | - | Y | Y | Y | Y | - | - | - | Y | Y | 6 |
| 2.5.1.32 | Y | Y | Y | Y | Y | Y | Y | - | - | Y | 8 |
| 2.5.1.54 | - | - | - | Y | - | - | - | - | - | - | 1 |
| 2.5.1.55 | - | - | - | Y | - | - | - | - | - | - | 1 |
| 2.5.1.61 | - | - | - | Y | - | - | - | - | - | - | 1 |
| 2.6.1.- | - | - | - | Y | Y | - | Y | - | - | - | 3 |
| 2.6.1.1 | Y | Y | Y | Y | Y | Y | Y | Y | Y | Y | 10 |
| 2.6.1.11 | Y | Y | - | - | Y | - | - | Y | - | - | 4 |
| 2.6.1.17 | Y | Y | - | - | Y | - | - | Y | - | - | 4 |
| 2.6.1.18 | - | - | - | Y | Y | - | - | - | - | - | 2 |
| 2.6.1.50 | - | - | - | Y | - | - | - | - | - | - | 1 |
| 2.6.1.52 | Y | Y | Y | Y | Y | Y | - | - | - | - | 6 |
| 2.6.1.62 | - | - | - | Y | - | - | - | - | - | - | 1 |
| 2.6.1.7 | Y | Y | Y | Y | Y | Y | Y | Y | Y | Y | 10 |
| 2.6.1.76 | - | - | - | Y | - | - | - | - | - | - | 1 |
| 2.6.1.9 | - | - | - | - | Y | - | - | - | - | - | 1 |
| 2.6.99.2 | - | - | - | Y | - | - | - | - | - | - | 1 |
| 2.7.1.- | Y | Y | Y | Y | Y | Y | Y | Y | Y | Y | 10 |
| 2.7.1.105 | Y | Y | Y | Y | Y | Y | Y | Y | - | Y | 9 |
| 2.7.1.108 | Y | Y | Y | Y | Y | Y | Y | Y | Y | Y | 10 |
| 2.7.1.130 | - | - | - | Y | Y | - | - | - | - | - | 2 |
| 2.7.1.134 | - | - | - | - | - | - | - | - | - | Y | 1 |
| 2.7.1.14 | Y | Y | Y | Y | Y | Y | - | - | - | - | 6 |
| 2.7.1.140 | - | - | - | - | - | - | - | - | Y | - | 1 |
| 2.7.1.149 | Y | Y | Y | Y | Y | Y | Y | Y | Y | Y | 10 |
| 2.7.1.15 | Y | Y | Y | Y | Y | Y | Y | Y | Y | Y | 10 |
| 2.7.1.151 | - | Y | Y | Y | Y | Y | Y | Y | Y | Y | 9 |
| 2.7.1.154 | Y | Y | Y | Y | Y | Y | Y | Y | Y | - | 9 |
| 2.7.1.159 | - | - | - | - | - | - | - | - | - | Y | 1 |
| 2.7.1.21 | Y | Y | Y | Y | Y | Y | Y | - | - | - | 7 |
| 2.7.1.36 | Y | Y | Y | Y | Y | Y | Y | Y | Y | - | 9 |
| 2.7.1.39 | - | - | - | Y | - | - | - | - | - | - | 1 |
| 2.7.1.40 | Y | Y | Y | Y | Y | Y | Y | Y | Y | Y | 10 |
| 2.7.1.48 | Y | Y | Y | Y | Y | Y | Y | Y | Y | Y | 10 |
| 2.7.1.69 | - | - | - | Y | - | - | - | - | - | - | 1 |
| 2.7.1.91 | Y | - | Y | Y | - | Y | - | Y | Y | Y | 7 |
| 2.7.2.11 | Y | Y | Y | - | Y | - | - | Y | - | Y | 6 |
| 2.7.2.4 | - | - | - | Y | - | - | - | Y | - | - | 2 |
| 2.7.2.8 | - | - | - | - | Y | - | - | - | - | - | 1 |
| 2.7.3.2 | - | - | - | - | - | Y | - | - | Y | Y | 3 |
| 2.7.3.3 | Y | Y | Y | Y | Y | Y | Y | Y | Y | Y | 10 |
| 2.7.4.2 | - | Y | Y | Y | Y | - | Y | Y | Y | Y | 8 |
| 2.7.4.6 | Y | Y | Y | Y | Y | Y | Y | Y | Y | Y | 10 |
| 2.7.6.3 | Y | Y | Y | Y | Y | Y | Y | Y | Y | Y | 10 |
| 2.7.7.- | Y | Y | Y | Y | Y | Y | Y | Y | Y | Y | 10 |
| 2.7.7.1 | Y | Y | Y | Y | Y | Y | Y | Y | Y | Y | 10 |
| 2.7.7.14 | Y | Y | Y | Y | Y | Y | Y | Y | Y | - | 9 |
| 2.7.7.15 | Y | Y | Y | Y | Y | Y | Y | Y | Y | Y | 10 |
| 2.7.7.23 | Y | Y | Y | Y | Y | Y | Y | Y | Y | Y | 10 |
| 2.7.7.24 | - | - | - | Y | - | - | - | - | - | - | 1 |
| 2.7.7.27 | Y | Y | Y | Y | Y | Y | Y | - | Y | Y | 9 |
| 2.7.7.30 | Y | Y | Y | Y | Y | - | - | - | - | - | 5 |
| 2.7.7.33 | - | - | - | Y | - | - | - | - | - | - | 1 |
| 2.7.7.38 | - | - | - | - | Y | - | - | - | - | - | 1 |
| 2.7.7.4 | Y | Y | Y | Y | Y | Y | Y | Y | Y | Y | 10 |
| 2.7.7.41 | Y | Y | Y | Y | Y | Y | Y | Y | Y | Y | 10 |
| 2.7.7.60 | - | - | - | - | Y | - | - | - | - | - | 1 |
| 2.7.7.62 | - | - | - | - | Y | - | - | - | - | - | 1 |
| 2.7.8.- | Y | Y | Y | Y | Y | Y | Y | Y | Y | Y | 10 |
| 2.7.8.1 | Y | Y | Y | Y | Y | Y | Y | Y | Y | Y | 10 |
| 2.7.8.13 | - | - | - | Y | - | - | - | - | - | - | 1 |
| 2.7.8.2 | Y | - | - | - | Y | - | - | Y | - | Y | 4 |
| 2.7.8.26 | - | - | - | Y | - | - | - | - | - | - | 1 |
| 2.7.8.5 | Y | Y | Y | Y | Y | Y | Y | Y | Y | Y | 10 |
| 2.7.9.3 | Y | Y | Y | Y | Y | Y | - | - | Y | Y | 8 |
| 2.8.1.6 | - | - | - | Y | - | - | Y | Y | - | - | 3 |
| 2.8.1.7 | Y | Y | Y | Y | Y | Y | Y | Y | Y | Y | 10 |
| 2.8.1.8 | Y | Y | Y | Y | Y | Y | Y | - | Y | Y | 9 |
| 2.8.2.- | Y | Y | Y | Y | Y | Y | Y | Y | Y | Y | 10 |
| 2.8.2.2 | Y | Y | Y | Y | Y | Y | Y | Y | Y | - | 9 |
| 2.8.3.- | Y | Y | Y | Y | Y | - | Y | - | Y | Y | 8 |
| 2.8.3.6 | - | - | - | - | Y | - | - | - | - | - | 1 |
| 2.8.4.1 | Y | Y | Y | Y | Y | Y | Y | Y | Y | Y | 10 |
| 3.1.1.- | Y | Y | Y | Y | Y | Y | Y | Y | Y | Y | 10 |
| 3.1.1.1 | Y | Y | Y | Y | Y | Y | Y | Y | Y | Y | 10 |
| 3.1.1.17 | Y | Y | Y | Y | Y | Y | Y | Y | Y | Y | 10 |
| 3.1.1.2 | Y | Y | Y | Y | Y | Y | - | - | - | - | 6 |
| 3.1.1.31 | Y | Y | Y | Y | Y | Y | Y | Y | Y | Y | 10 |
| 3.1.1.32 | Y | Y | Y | Y | Y | Y | Y | Y | Y | - | 9 |
| 3.1.1.4 | Y | Y | Y | Y | Y | Y | Y | Y | Y | Y | 10 |
| 3.1.1.45 | Y | Y | Y | Y | Y | Y | Y | - | - | - | 7 |
| 3.1.1.47 | Y | Y | Y | Y | Y | Y | Y | Y | Y | Y | 10 |
| 3.1.1.7 | Y | Y | Y | Y | Y | Y | Y | Y | Y | Y | 10 |
| 3.1.2.- | Y | Y | Y | Y | Y | Y | Y | Y | Y | Y | 10 |
| 3.1.2.14 | - | - | - | Y | Y | - | - | - | Y | - | 3 |
| 3.1.2.2 | Y | Y | Y | Y | Y | Y | - | - | - | Y | 7 |
| 3.1.2.4 | Y | Y | Y | Y | Y | Y | Y | - | Y | Y | 9 |
| 3.1.3.- | Y | Y | Y | Y | Y | Y | Y | Y | Y | Y | 10 |
| 3.1.3.1 | Y | Y | Y | Y | Y | Y | - | Y | Y | - | 8 |
| 3.1.3.11 | Y | Y | Y | Y | Y | Y | Y | Y | Y | Y | 10 |
| 3.1.3.18 | Y | Y | Y | Y | Y | Y | Y | Y | Y | Y | 10 |
| 3.1.3.25 | Y | Y | Y | Y | Y | Y | Y | Y | Y | Y | 10 |
| 3.1.3.26 | - | - | - | Y | Y | - | - | - | - | - | 2 |
| 3.1.3.27 | - | - | - | Y | Y | - | - | - | - | - | 2 |
| 3.1.3.4 | Y | Y | Y | Y | Y | Y | Y | Y | Y | Y | 10 |
| 3.1.3.45 | - | - | - | - | Y | - | - | - | - | - | 1 |
| 3.1.3.5 | Y | Y | Y | Y | Y | Y | Y | Y | Y | Y | 10 |
| 3.1.3.56 | Y | Y | Y | Y | Y | Y | Y | Y | Y | Y | 10 |
| 3.1.3.66 | Y | Y | Y | Y | Y | Y | Y | Y | Y | - | 9 |
| 3.1.3.67 | Y | Y | Y | Y | Y | Y | Y | Y | Y | Y | 10 |
| 3.1.3.77 | Y | Y | Y | Y | Y | Y | Y | Y | - | - | 8 |
| 3.1.3.8 | - | - | - | Y | - | - | - | - | - | - | 1 |
| 3.1.4.- | Y | Y | Y | Y | Y | Y | Y | Y | Y | Y | 10 |
| 3.1.4.17 | Y | Y | Y | Y | Y | Y | Y | Y | Y | Y | 10 |
| 3.1.6.1 | Y | Y | Y | Y | Y | Y | Y | Y | Y | - | 9 |
| 3.1.6.2 | - | - | - | - | - | Y | - | - | - | - | 1 |
| 3.1.7.2 | Y | Y | Y | Y | Y | Y | Y | Y | Y | - | 9 |
| 3.1.8.1 | Y | Y | Y | Y | Y | Y | - | - | - | - | 6 |
| 3.11.1.2 | - | - | - | - | Y | - | - | - | - | - | 1 |
| 3.2.1.- | Y | Y | Y | Y | Y | Y | Y | Y | Y | Y | 10 |
| 3.2.1.1 | Y | Y | Y | Y | Y | Y | - | - | - | Y | 7 |
| 3.2.1.10 | - | - | - | - | Y | - | Y | - | Y | Y | 4 |
| 3.2.1.14 | Y | Y | Y | Y | Y | Y | Y | Y | Y | Y | 10 |
| 3.2.1.21 | Y | Y | Y | Y | Y | Y | - | - | - | - | 6 |
| 3.2.1.22 | - | Y | - | - | - | Y | - | - | Y | - | 3 |
| 3.2.1.23 | Y | Y | Y | Y | Y | Y | Y | Y | - | Y | 9 |
| 3.2.1.26 | - | - | - | - | - | - | Y | Y | - | - | 2 |
| 3.2.1.3 | Y | Y | Y | Y | Y | Y | Y | Y | Y | Y | 10 |
| 3.2.1.31 | Y | Y | Y | Y | Y | Y | Y | Y | Y | Y | 10 |
| 3.2.1.52 | Y | Y | Y | Y | Y | Y | Y | Y | Y | Y | 10 |
| 3.2.2.9 | - | - | - | Y | - | - | - | - | - | - | 1 |
| 3.3.2.1 | Y | Y | Y | Y | Y | Y | Y | Y | Y | Y | 10 |
| 3.3.2.10 | Y | Y | Y | - | Y | - | - | - | - | - | 4 |
| 3.3.2.6 | Y | Y | Y | Y | Y | Y | Y | Y | Y | - | 9 |
| 3.3.2.9 | Y | Y | Y | Y | Y | Y | Y | Y | Y | Y | 10 |
| 3.4.-.- | Y | Y | Y | Y | Y | Y | Y | Y | Y | Y | 10 |
| 3.4.11.1 | Y | Y | Y | Y | Y | Y | Y | Y | Y | Y | 10 |
| 3.5.1.- | Y | Y | Y | Y | Y | Y | Y | Y | Y | Y | 10 |
| 3.5.1.1 | Y | Y | Y | Y | Y | Y | Y | Y | Y | - | 9 |
| 3.5.1.16 | Y | Y | Y | Y | Y | Y | - | - | Y | - | 7 |
| 3.5.1.23 | Y | Y | Y | Y | Y | Y | - | - | Y | Y | 8 |
| 3.5.1.24 | - | - | - | Y | - | - | - | - | - | - | 1 |
| 3.5.1.25 | Y | Y | Y | Y | Y | Y | Y | - | - | Y | 8 |
| 3.5.1.32 | - | - | - | Y | - | - | - | - | - | - | 1 |
| 3.5.1.4 | Y | Y | Y | Y | Y | Y | Y | Y | Y | Y | 10 |
| 3.5.1.41 | Y | Y | Y | Y | Y | Y | - | - | Y | Y | 8 |
| 3.5.1.6 | Y | Y | Y | Y | Y | Y | Y | Y | - | - | 8 |
| 3.5.1.87 | - | - | - | Y | - | - | - | - | - | - | 1 |
| 3.5.1.9 | Y | Y | Y | Y | Y | Y | - | - | Y | Y | 8 |
| 3.5.2.14 | - | - | - | Y | Y | - | - | - | - | - | 2 |
| 3.5.2.17 | Y | Y | Y | Y | Y | - | - | - | - | - | 5 |
| 3.5.2.2 | Y | Y | Y | Y | Y | Y | Y | Y | Y | Y | 10 |
| 3.5.2.6 | Y | Y | Y | Y | Y | Y | Y | - | Y | Y | 9 |
| 3.5.2.9 | Y | Y | Y | Y | Y | Y | Y | Y | - | - | 8 |
| 3.5.4.- | Y | Y | Y | Y | Y | - | - | Y | Y | Y | 8 |
| 3.5.4.1 | - | - | - | Y | - | - | Y | - | Y | - | 3 |
| 3.5.4.16 | Y | Y | Y | Y | Y | Y | Y | - | Y | Y | 9 |
| 3.5.4.25 | - | - | - | - | - | Y | - | Y | - | - | 2 |
| 3.5.4.26 | Y | Y | Y | Y | Y | - | Y | Y | - | Y | 8 |
| 3.5.4.4 | Y | Y | Y | Y | Y | Y | - | - | Y | Y | 8 |
| 3.5.4.5 | Y | Y | Y | Y | Y | Y | Y | Y | Y | Y | 10 |
| 3.5.5.1 | Y | Y | Y | Y | Y | Y | Y | Y | Y | Y | 10 |
| 3.6.1.- | Y | Y | Y | Y | Y | Y | Y | Y | Y | Y | 10 |
| 3.6.1.11 | - | - | - | Y | - | - | - | - | - | - | 1 |
| 3.6.1.14 | Y | Y | Y | Y | Y | Y | Y | Y | Y | Y | 10 |
| 3.6.1.17 | Y | Y | Y | Y | Y | Y | Y | Y | Y | Y | 10 |
| 3.6.1.19 | Y | Y | Y | Y | Y | Y | Y | Y | Y | Y | 10 |
| 3.6.1.28 | Y | Y | Y | Y | Y | Y | Y | Y | Y | Y | 10 |
| 3.6.1.29 | Y | Y | Y | Y | Y | Y | Y | Y | Y | Y | 10 |
| 3.7.1.- | - | - | - | Y | - | - | - | - | - | - | 1 |
| 3.7.1.2 | Y | Y | Y | Y | Y | Y | Y | - | - | - | 7 |
| 3.7.1.3 | Y | Y | Y | Y | Y | Y | Y | - | Y | Y | 9 |
| 4.1.1.- | - | - | - | Y | - | - | - | - | - | - | 1 |
| 4.1.1.22 | - | - | Y | Y | - | - | - | - | - | - | 2 |
| 4.1.1.25 | - | - | - | Y | - | - | - | - | - | - | 1 |
| 4.1.1.28 | Y | Y | Y | Y | Y | Y | Y | Y | Y | Y | 10 |
| 4.1.1.33 | Y | Y | Y | Y | Y | Y | Y | - | Y | Y | 9 |
| 4.1.1.36 | Y | Y | Y | Y | Y | Y | Y | Y | Y | Y | 10 |
| 4.1.1.44 | - | - | - | Y | - | - | - | - | - | - | 1 |
| 4.1.1.45 | Y | Y | Y | Y | Y | Y | - | - | - | - | 6 |
| 4.1.1.50 | Y | Y | Y | Y | Y | Y | Y | - | Y | - | 8 |
| 4.1.1.68 | - | - | - | Y | - | - | - | - | - | - | 1 |
| 4.1.1.8 | Y | - | - | - | - | Y | - | - | - | - | 2 |
| 4.1.2.- | Y | Y | Y | Y | Y | - | Y | Y | Y | Y | 9 |
| 4.1.2.13 | Y | Y | Y | Y | Y | Y | Y | Y | Y | Y | 10 |
| 4.1.2.27 | Y | Y | Y | Y | Y | Y | Y | Y | Y | Y | 10 |
| 4.1.2.5 | Y | Y | Y | Y | Y | Y | Y | - | - | - | 7 |
| 4.1.3.- | - | - | - | - | Y | - | - | - | - | - | 1 |
| 4.1.3.16 | - | - | - | Y | - | - | - | - | - | - | 1 |
| 4.1.3.17 | - | - | - | Y | - | - | - | - | - | - | 1 |
| 4.1.3.39 | - | - | - | - | Y | - | - | - | - | - | 1 |
| 4.1.3.4 | Y | Y | Y | Y | Y | Y | Y | Y | - | - | 8 |
| 4.2.-.- | Y | Y | Y | Y | Y | Y | Y | Y | - | Y | 9 |
| 4.2.1.- | Y | Y | Y | Y | Y | Y | Y | Y | Y | - | 9 |
| 4.2.1.1 | Y | Y | Y | Y | Y | Y | Y | Y | Y | Y | 10 |
| 4.2.1.104 | - | - | - | - | - | - | Y | Y | Y | Y | 4 |
| 4.2.1.107 | Y | Y | Y | Y | Y | Y | - | Y | Y | - | 8 |
| 4.2.1.109 | - | - | Y | Y | Y | - | - | Y | - | - | 4 |
| 4.2.1.17 | Y | Y | Y | Y | Y | Y | Y | Y | Y | Y | 10 |
| 4.2.1.18 | - | - | - | - | - | Y | - | Y | - | - | 2 |
| 4.2.1.3 | Y | Y | Y | Y | Y | Y | Y | Y | Y | Y | 10 |
| 4.2.1.33 | - | - | - | Y | - | - | - | - | - | - | 1 |
| 4.2.1.42 | - | - | - | Y | - | - | - | - | - | - | 1 |
| 4.2.1.44 | - | - | - | Y | - | - | - | - | - | - | 1 |
| 4.2.1.46 | Y | Y | Y | Y | Y | Y | Y | Y | Y | Y | 10 |
| 4.2.1.49 | Y | Y | Y | Y | Y | Y | Y | Y | - | - | 8 |
| 4.2.1.6 | - | - | - | Y | - | - | - | - | - | - | 1 |
| 4.2.1.70 | Y | Y | Y | Y | Y | Y | Y | Y | Y | Y | 10 |
| 4.2.1.8 | - | - | - | Y | - | - | - | - | - | - | 1 |
| 4.2.1.9 | Y | Y | Y | Y | Y | Y | Y | Y | Y | Y | 10 |
| 4.2.2.2 | - | - | - | - | - | - | Y | Y | - | - | 2 |
| 4.2.3.1 | Y | - | - | Y | - | Y | Y | Y | Y | - | 6 |
| 4.2.3.4 | - | - | - | Y | - | - | - | - | - | - | 1 |
| 4.3.1.3 | Y | Y | Y | Y | Y | Y | Y | Y | - | - | 8 |
| 4.3.2.1 | - | - | - | - | Y | - | - | - | - | - | 1 |
| 4.3.2.2 | Y | Y | Y | Y | Y | Y | Y | Y | Y | Y | 10 |
| 4.3.3.2 | Y | Y | Y | Y | Y | Y | - | Y | Y | - | 8 |
| 4.4.1.- | - | - | - | - | Y | - | - | - | - | - | 1 |
| 4.4.1.1 | Y | Y | Y | Y | Y | Y | Y | - | Y | Y | 9 |
| 4.4.1.11 | Y | Y | Y | Y | Y | - | - | - | - | - | 5 |
| 4.4.1.15 | - | - | - | - | Y | - | - | - | - | - | 1 |
| 4.4.1.16 | Y | Y | Y | Y | Y | Y | - | - | Y | Y | 8 |
| 4.4.1.17 | Y | Y | Y | Y | Y | Y | Y | Y | Y | Y | 10 |
| 4.4.1.22 | - | - | - | Y | - | - | - | - | - | - | 1 |
| 4.4.1.5 | - | - | - | Y | - | Y | Y | Y | Y | Y | 6 |
| 4.4.1.8 | - | - | - | Y | Y | - | - | - | - | - | 2 |
| 4.6.1.1 | Y | Y | Y | Y | Y | Y | Y | Y | Y | Y | 10 |
| 4.6.1.12 | - | - | - | - | Y | - | - | - | - | - | 1 |
| 4.99.1.1 | Y | Y | Y | Y | Y | Y | Y | Y | Y | Y | 10 |
| 4.99.1.3 | - | - | - | - | - | - | - | - | Y | - | 1 |
| 4.99.1.4 | - | - | - | Y | - | - | - | - | - | - | 1 |
| 5.1.-.- | Y | Y | Y | Y | Y | Y | - | - | - | - | 6 |
| 5.1.1.4 | - | - | - | Y | - | - | - | - | - | - | 1 |
| 5.1.2.2 | - | - | - | Y | - | - | - | - | - | - | 1 |
| 5.1.3.- | - | - | - | Y | - | - | - | - | - | - | 1 |
| 5.1.3.13 | Y | Y | Y | Y | Y | Y | Y | - | Y | - | 8 |
| 5.1.99.4 | Y | Y | Y | Y | Y | Y | Y | - | - | - | 7 |
| 5.2.1.2 | Y | Y | Y | Y | Y | Y | - | - | - | - | 6 |
| 5.3.1.16 | - | - | - | - | Y | - | - | - | - | - | 1 |
| 5.3.1.23 | Y | Y | Y | Y | Y | Y | Y | Y | Y | Y | 10 |
| 5.3.2.1 | Y | Y | Y | Y | Y | Y | Y | Y | Y | Y | 10 |
| 5.3.3.- | Y | Y | Y | Y | Y | Y | Y | - | Y | Y | 9 |
| 5.3.3.12 | - | - | - | - | - | - | - | - | - | Y | 1 |
| 5.3.3.5 | Y | Y | Y | - | - | Y | Y | Y | - | Y | 7 |
| 5.3.3.6 | - | - | - | Y | Y | - | - | - | - | - | 2 |
| 5.3.3.8 | Y | Y | Y | Y | Y | Y | Y | Y | Y | Y | 10 |
| 5.3.99.2 | Y | Y | Y | - | - | Y | - | - | - | - | 4 |
| 5.3.99.3 | Y | Y | Y | Y | Y | Y | Y | Y | Y | - | 9 |
| 5.3.99.5 | Y | Y | Y | Y | Y | Y | - | Y | - | Y | 8 |
| 5.4.1.2 | - | - | - | Y | Y | - | - | - | - | - | 2 |
| 5.4.2.1 | Y | Y | Y | Y | Y | Y | Y | Y | Y | Y | 10 |
| 5.4.2.10 | - | - | - | - | Y | - | - | - | - | - | 1 |
| 5.4.3.8 | - | - | - | Y | - | - | - | - | - | - | 1 |
| 5.4.99.2 | Y | Y | Y | Y | Y | Y | - | - | - | Y | 7 |
| 5.5.1.1 | - | - | - | Y | - | - | - | - | - | - | 1 |
| 5.5.1.7 | Y | Y | Y | Y | Y | Y | - | - | Y | Y | 8 |
| 6.1.1.1 | Y | Y | Y | Y | Y | Y | Y | Y | Y | Y | 10 |
| 6.1.1.10 | Y | Y | Y | Y | Y | Y | Y | Y | Y | Y | 10 |
| 6.1.1.11 | Y | Y | Y | Y | Y | Y | Y | Y | Y | Y | 10 |
| 6.1.1.12 | Y | Y | Y | Y | Y | Y | Y | Y | Y | Y | 10 |
| 6.1.1.13 | - | - | Y | - | - | Y | - | - | - | - | 2 |
| 6.1.1.14 | Y | Y | Y | Y | Y | Y | Y | Y | Y | Y | 10 |
| 6.1.1.15 | Y | Y | Y | Y | Y | Y | Y | Y | Y | Y | 10 |
| 6.1.1.17 | Y | Y | Y | Y | Y | Y | Y | Y | Y | Y | 10 |
| 6.1.1.18 | Y | Y | Y | Y | Y | Y | Y | - | Y | Y | 9 |
| 6.1.1.19 | Y | Y | Y | Y | Y | Y | Y | Y | Y | Y | 10 |
| 6.1.1.2 | Y | Y | Y | Y | Y | Y | Y | Y | Y | Y | 10 |
| 6.1.1.20 | Y | Y | Y | Y | Y | Y | Y | Y | Y | Y | 10 |
| 6.1.1.21 | Y | Y | Y | Y | Y | Y | Y | Y | Y | Y | 10 |
| 6.1.1.22 | Y | Y | Y | Y | Y | Y | Y | Y | Y | Y | 10 |
| 6.1.1.3 | Y | Y | Y | Y | Y | Y | Y | Y | Y | Y | 10 |
| 6.1.1.4 | Y | Y | Y | Y | Y | Y | Y | Y | Y | Y | 10 |
| 6.1.1.5 | Y | Y | Y | Y | Y | Y | Y | Y | Y | Y | 10 |
| 6.1.1.6 | Y | Y | Y | Y | Y | Y | Y | Y | Y | Y | 10 |
| 6.1.1.7 | Y | Y | Y | Y | Y | Y | Y | Y | Y | Y | 10 |
| 6.1.1.9 | Y | Y | Y | Y | Y | Y | Y | Y | Y | Y | 10 |
| 6.2.1.- | Y | Y | Y | Y | Y | Y | Y | Y | Y | Y | 10 |
| 6.2.1.1 | Y | Y | Y | Y | Y | Y | Y | Y | Y | Y | 10 |
| 6.2.1.12 | Y | Y | Y | Y | Y | Y | Y | Y | Y | Y | 10 |
| 6.2.1.20 | - | - | - | Y | - | - | - | - | - | - | 1 |
| 6.2.1.26 | Y | Y | Y | Y | Y | Y | Y | - | Y | - | 8 |
| 6.3.1.11 | - | - | - | Y | - | - | - | - | - | - | 1 |
| 6.3.2.- | Y | Y | Y | Y | Y | Y | Y | Y | Y | Y | 10 |
| 6.3.2.13 | - | - | - | Y | - | - | - | - | - | - | 1 |
| 6.3.2.17 | Y | Y | Y | Y | Y | Y | Y | - | Y | Y | 9 |
| 6.3.2.2 | Y | Y | Y | Y | Y | Y | Y | Y | Y | Y | 10 |
| 6.3.2.26 | - | - | Y | - | - | Y | - | - | - | - | 2 |
| 6.3.2.4 | - | - | - | - | Y | - | - | - | - | - | 1 |
| 6.3.2.6 | Y | Y | Y | Y | Y | - | - | - | - | Y | 6 |
| 6.3.3.1 | Y | Y | Y | Y | Y | - | - | - | - | Y | 6 |
| 6.3.3.3 | - | - | - | Y | Y | - | - | - | - | - | 2 |
| 6.3.4.13 | Y | Y | Y | Y | Y | - | - | - | - | Y | 6 |
| 6.3.4.4 | Y | Y | Y | Y | Y | Y | Y | Y | Y | Y | 10 |
| 6.3.4.5 | - | - | - | - | Y | - | - | - | - | - | 1 |
| 6.3.4.9 | Y | Y | Y | Y | Y | Y | Y | - | Y | Y | 9 |
| 6.3.5.- | - | - | - | Y | - | Y | - | Y | - | - | 3 |
| 6.3.5.2 | Y | Y | Y | Y | Y | Y | Y | - | Y | Y | 9 |
| 6.3.5.3 | Y | Y | Y | Y | Y | - | - | - | - | Y | 6 |
| 6.3.5.6 | Y | Y | Y | Y | Y | Y | Y | Y | Y | Y | 10 |
| 6.3.5.7 | Y | Y | Y | Y | Y | Y | Y | Y | Y | Y | 10 |
| 6.4.1.2 | Y | Y | Y | Y | Y | Y | Y | Y | Y | Y | 10 |
|  |  |  |  |  |  |  |  |  |  |  |  |

| **Table S3. Insecticides in DrugBank.** Chokepoint drugs are listed in bold. | | | | | |  |  |  |  |
| --- | --- | --- | --- | --- | --- | --- | --- | --- | --- |
| **DrugBank #** | **Name** | **EC** | **Chkpt?** | **NemSet?** | **Metabolic**  **Pathways (#num)** |  |  |  |  |
| DB00431 | Lindane | NoEC | N |  |  |  |  |  |  |
| **DB00572** | Atropine | 3.1.1.4 | Y | Dros | Lipid (1) |  |  |  |  |
|  |  | 3.1.1.7 | Y | Dros | Lipid (1) |  |  |  |  |
| DB00648 | Mitotane | 2.7.7.7 | N | Dros | Nucleotide (1) |  |  |  |  |
| DB00676 | Benzyl Benzoate | NoEC | N |  |  |  |  |  |  |
| **DB00772** | Malathion | 3.1.1.7 | Y | Dros | Lipid (1) |  |  |  |  |
|  |  | 3.1.1.8 | N | Dros |  |  |  |  |  |
| DB02775 | Benzoic Acid Phenylmethylester | NoEC | N |  |  |  |  |  |  |
| **DB03703** | Cyclohexanol | 1.1.1.1 | Y | Dros |  |  |  |  |  |
| **DB04628** | Allosamidin | 3.2.1.14 | Y | Dros | Carbohydrate (1) |  |  |  |  |
|  |  | 1.14.15.4 | Y |  | Lipid (1) |  |  |  |  |
|  |  | 1.14.15.5 | N |  |  |  |  |  |  |
|  |  | 2.7.11.1 | N | Dros |  |  |  |  |  |
| DB04930 | Permethrin | NoEC | N |  |  |  |  |  |  |

**Table S4. Antiparasitic Drugs in DrugBank^a^.**

| **DrugBank ID** | **Name** | **EC** | **Chkpt** | **NemSet^b^** | **Metabolic**  **Pathways (num)^C^** |
| --- | --- | --- | --- | --- | --- |
| DB00205 | Pyrimethamine | 1.5.1.3 | N |  | Cofactors and Vitamins (2) |
|  |  | 2.1.1.45 | N |  | Nucleotide (2) |
| **DB00468** | Quinine | 3.1.1.4 | Y | CommNem | Lipid (1) |
| **DB00608** | Chloroquine | 2.5.1.18 | Y | CommNem | Other Amino Acids (1) |
|  |  | 2.7.7.7 | N |  | Nucleotide (2) |
| **DB00613** | Amodiquine | 2.1.1.8 | Y |  | Amino Acid (1) |
| **DB00711** | Diethylcarbamazine | 1.9.3.1 | N |  | Energy (1) |
|  |  | 1.13.11.34 | Y |  | Carbohydrate (1) |
| DB00730 | Thiabendazole | 1.3.99.1 | N |  | Carbohydrate, Xenobiotics, Energy (5) |
| **DB00848** | Levamisole | 6.1.1.16 | N |  | Amino Acid (1) |
|  |  | 3.1.3.1 | Y | UniNem | Xenobiotics, Lipid, Cofactors (5) |
| **DB01058** | Praziquantel | 2.5.1.18 | Y | CommNem |  |
| **DB01103** | Quinacrine | 3.6.3.44 | N |  |  |
|  |  | 3.1.1.4 | Y | CommNem | Lipid (1) |
|  |  | 3.1.1.5 | N |  |  |
|  |  | 3.1.4.11 | N |  | Carbohydrate (1) |
|  |  | 1.13.11.34 | Y |  | Carbohydrate (1) |
| **DB01117** | Atovaquone | 1.3.3.1 | N |  | Nucleotide (1) |
|  |  | 1.3.5.2 | Y | UniNem |  |
|  |  | 3.4.24.64 | N |  |  |
| DB01131 | Proguanil | 2.1.1.45 | N |  | Nucleotide, Cofactors (2) |
|  |  | 1.5.1.3 | N |  | Cofactors (2) |
| DB01218 | Halofantrine | 2.7.11.1 | N |  |  |
|  |  | 3.6.3.14 | N |  | Energy (1) |
| **DB02962** | Benzimidazole | 1.11.1.5 | N |  |  |
|  |  | 2.4.2.21 | Y | UniNem | Cofactors (2) |
| **DB04786** | Suramin | 3.1.1.4 | Y | CommNem | Lipid (1) |
| **DB05630** | Na stiboguconate | 5.99.1.2 | N |  |  |
|  |  | 3.5.1.- | Y | CommNem |  |
|  |  | 3.4.21.5 | N |  |  |
|  |  | 4.2.1.2 | N |  | Carbohydrate (2) |

**^a^** Chokepoint drugs are listed in bold. The following are antiparasitic compounds in DrugBank with which no ECs are associated: Hydroxychloroquine (DB01611), Primaquine (DB01087), Berberine (DB04115), Mefloquine (DB00358), Albendazole (DB00518), Mebendazole(DB00643), Fuzazolidone (DB00614), Oxamniquine (DB01096), Benzyl Benzoate (DB00676), ivermectin (DB00602), Piperazine (DB00592), and Lindane (DB00431).

**^b^** CommNem – the intersection of ECs for all 10 nematode species and UniNem – the union of ECs for all 10 nematode species. All CommNem targets are a subset of UniNem.

**^c^**Number of major metabolic pathways in which the chokepoint is found

**Table S5: KEGG drugs targets that hit nematode enzymes** ^a^**.**

| **EC Number** | **Group^b^** | **Action on Target** | **Description** |
| --- | --- | --- | --- |
| 1.1.1.145 | UniNem | 3beta-hydroxysteroid dehydrogenase inhibitor | Adrenocortical suppressant |
| 1.1.1.205 | CommNem | IMP dehydrogenase inhibitor | Immunosuppressant, antiparasitic |
| 1.1.1.21 | UniNem | aldose reductase inhibitor | Antidiabetic |
| 1.14.14.1 | CommNem | aromatase inhibitor | Antineoplastic |
| 1.14.16.2 | UniNem | tyrosine 3-monooxygenase inhibitor | Antihypertensive |
| 1.14.17.1 | CommNem | dopamine beta-hydroxylase inhibitor | Treatment of congestive heart failure |
| 1.2.1.3 | UniNem | aldehyde dehydrogenase inhibitor | Alcohol deterrent |
| 1.3.1.- | CommNem | dihydropyrimidine dehydrogenase inhibitor | Antineoplastic |
| 1.3.99.5 | UniNem | 5alpha-reductase inhibitor | Inhibitor [alpha reductase], Treatment of prostate cancers, Antineoplastic which acts as an antiproliferative, topoisomerase II |
| 1.4.3.4 | UniNem | monoamine oxidase B (MAO-B) inhibitor | Antibacterial, Antiviral, Antiparkinsonian, Antidepressant, Antituberculous, Antimalarial, Antihypertensive, Anticonvulsant, Antidepressant, Antiprotozoal, |
| 1.8.1.9 | UniNem | thioredoxin reductase inhibitor | Anti-acne |
| 2.3.1.21 | CommNem | carnitine palmitoyltransferase (CPT)-1 inhibitor | Vasodilator |
| 2.3.1.26 | UniNem | cholesterol acyltransferase (ACAT) inhibitor | Anti-atherosclerotic, Antihyperlipidemic |
| 2.3.1.85 | UniNem | fatty acid synthase inhibitor | Antibacterial |
| 2.3.2.2 | CommNem | gamma-glutamyltransferase inhibitor | Antineoplastic |
| 2.4.1.17 | CommNem | uridine diphosphoglucuronosyltransferase (UGT) I A1 | Antiviral, HIV protease inihibitor |
| 2.5.1.15 | UniNem | dihydropteroate synthase inhibitor | --------- |
| 3.1.1.4 | CommNem | phospholipase A2 (PLA2) inhibitor | Anti-inflammatory; Antimalarial; Antiprotozoal |
| 3.1.1.7 | CommNem | acetylcholinesterase (AChE) inhibitor | Cholinergic; Alzheimer's disease treatment [adjunct]; Antidepressant; Anthelmintic |
| 3.1.4.17 | CommNem | phosphodiesterase (PDE) III inhibitor | Relaxant [smooth muscle]; Cardiotonic; Treatment of asthma, COPD, arthritis, atopic dermatitis; Vasodilator |
| 3.2.1.1 | UniNem | alpha-amylase inhibitor | Inhibitor [alpha-glucosidase] |
| 3.2.1.22 | UniNem | Agalsidase alfa Agalsidase beta | Treatment of Fabry Disease |
| 3.5.2.6 | UniNem | beta-lactamase inhibitor | Inhibitor [beta-lactamase], Antibiotic |
| 4.1.1.22 | UniNem | histidine decarboxylase inhibitor | Inhibitor [histidine decarboxylase] |
| 4.2.1.1 | CommNem | carbonic anhydrase inhibitor | Antidiabetic; Antiglaucoma agent; Carbonic anhydrase inhibitor |
| 4.6.1.1 | CommNem | adenylate cyclase activator | Cardiotonic |
| 5.3.99.5 | UniNem | thromboxane A2 (TXA2) synthase inhibitor | Inhibitor [thromboxane synthetase], Antithrombotic |
| 6.1.1.5 | CommNem | isoleucine tRNA ligase inhibitor, ptotein synthesis inhibitor | Antibacterial |
| 6.3.2.4 | UniNem | D-alanine-D-alanine ligase inhibitor, cell wall synthesis inhibitor | Antibacterial [tuberculostatic] |

^a^ The corresponding drugs are listed in Supplementary Table 2 and 3.

^b^ All CommNem targets are a subset of UniNem.

**Table S6. KEGG drugs that hit Common Nematodes ECs.**

| **EC Number** | **Drug** | **Target** | **Activity** |
| --- | --- | --- | --- |
| 1.1.1.205 | D05095 D05094 D04936 D00752 | IMP dehydrogenase inhibitor | Immunosuppressant, antiparasitic |
| 1.14.14.1 | D07940 D03786 D03784 D03781 D03778 D03749 D02451 D00964 D00963 D00960 D00574 D00153 | aromatase inhibitor | Antineoplastic |
| 1.14.17.1 | D03787 | dopamine beta-hydroxylase inhibitor | Treatment of congestive heart failure |
| 1.3.1.- | D03998 | dihydropyrimidine dehydrogenase inhibitor | Antineoplastic |
| 2.3.1.21 | D05292 | carnitine palmitoyltransferase (CPT)-1 inhibitor | Vasodilator |
| 2.3.2.2 | D02755 | gamma-glutamyltransferase inhibitor | Antineoplastic |
| 2.4.1.17 | D07471 D06478 D05538 D03843 D03842 D03840 D03839 D03837 D03835 D03833 D03656 D03253 D02867 D02861 D02498 D02497 D01425 D01276 D01160 D00899 D00897 D00894 D00429 D00427 | uridine diphosphoglucuronosyltransferase (UGT) I A1 | Antiviral, HIV protease inihibitor |
| 3.1.1.4 | D08179 D08085 D07441 D07337 D05040 D05039 D05033 D04681 D04092 D03733 D03409 D03239 D03099 D02729 D02565 D02558 D02418 D02193 D02173 D02068 D01572 D01228 D01001 D00998 D00995 D00994 D00805 D00670 D00667 D00534 D00487 D00469 D00196 D00043 D02580 D02579 D02564 D02548 D02002 D01888 D01501 D01097 D00777 D00346 D06283 D03938 D03650 D02548 D02002 D01888 D01501 D01097 D00777 D00346 D02341 D00169 D00151 | phospholipase A2 (PLA2) inhibitor | Anti-inflammatory; Antimalarial; Antiprotozoal |
| 3.1.1.7 | D08555 D08261 D07869 D06481 D06288 D05590 D05215 D04292 D03826 D03822 D03791 D03751 D03239 D03099 D02729 D02565 D02558 D02418 D02193 D02173 D02068 D01572 D01228 D01001 D00998 D00995 D00994 D00805 D00670 D00667 D00534 D00487 D00469 D00196 D00043 | acetylcholinesterase (AChE) inhibitor | Cholinergic; Alzheimer's disease treatment [adjunct]; Antidepressant; Anthelmintic |
| 3.1.4.17 | D08668 D08514 D05744 D05429 D04720 D04628 D04529 D04508 D04185 D04004 D03657 D03516 D03260 D03217 D02229 D02085 D02084 D02042 D02008 D01771 D01704 D01690 D01133 D00417 D00231 D00227 | phosphodiesterase (PDE) III inhibitor | Relaxant [smooth muscle]; Cardiotonic; Treatment of asthma, COPD, arthritis, atopic dermatitis; Treatment of male erectile and female sexual dysfunction; Vasodilator |
| 4.2.1.1 | D03845 D00653 D00652 D00219 | carbonic anhydrase inhibitor | Antidiabetic; Antiglaucoma agent; Carbonic anhydrase inhibitor |
| 4.6.1.1 | D01697 | adenylate cyclase activator | Cardiotonic |
| 6.1.1.5 | D02195 D01076 | isoleucine tRNA ligase inhibitor, ptotein synthesis inhibitor | Antibacterial |

**Table S7. Additional KEGG drugs that hit from UniNem.**

| 1.1.1.145 | D01180 | 3beta-hydroxysteroid dehydrogenase inhibitor | Adrenocortical suppressant |
| --- | --- | --- | --- |
| 1.1.1.21 | D05893 D03807 D03806 D03805 D03803 D02835 D02328 D01842 D01715 D01688 | aldose reductase inhibitor | Antidiabetic |
| 1.14.16.2 | D00762 | tyrosine 3-monooxygenase inhibitor | Antihypertensive |
| 1.2.1.3 | D00131 D00123 | aldehyde dehydrogenase inhibitor | Alcohol deterrent |
| 1.3.99.5 | D04498 D03107 D01134 | 5alpha-reductase inhibitor | Inhibitor [alpha reductase], Treatment of prostate cancers, Antineoplastic which acts as an antiproliferative, topoisomerase II |
| 1.4.3.4 | D08469 D08179 D08085 D07441 D07337 D05040 D05039 D05033 D04681 D04092 D03733 D03409 D03239 D03099 D02729 D02565 D02558 D02418 D02193 D02173 D02068 D01572 D01228 D01001 D00998 D00995 D00994 D00805 D00670 D00667 D00534 D00487 D00469 D00196 D00043 D02580 D02579 D02564 D02548 D02002 D01888 D01501 D01097 D00777 D00346 | monoamine oxidase B (MAO-B) inhibitor | Antibacterial, Antiviral, Antiparkinsonian, Treatment of Alzheimer's disease, Antidepressant, Antituberculous, Antimalarial, Antihypertensive, Anticonvulsant, Psychotropic, Antidepressant, Antiprotozoal, Neuroprotective; |
| 1.8.1.9 | D03034 | thioredoxin reductase inhibitor | Anti-acne |
| 2.3.1.26 | D03735 D03734 D03012 | cholesterol acyltransferase (ACAT) inhibitor | Anti-atherosclerotic, Antihyperlipidemic |
| 2.3.1.85 | D00144 | fatty acid synthase inhibitor | Antibacterial |
| 2.5.1.15 | D02520 | dihydropteroate synthase inhibitor | --------- |
| 3.2.1.1 | D00216 | alpha-amylase inhibitor | Inhibitor [alpha-glucosidase] |
| 3.2.1.22 | D02784 D03228 | Agalsidase beta | Treatment of Fabry Disease |
| 3.5.2.6 | D03707 D02503 D00660 | beta-lactamase inhibitor | Inhibitor [beta-lactamase], Antibiotic |
| 4.1.1.22 | D03158 | histidine decarboxylase inhibitor | Inhibitor [histidine decarboxylase] |
| 5.3.99.5 | D05727 D05502 D04276 D03663 D03661 | thromboxane A2 (TXA2) synthase inhibitor | Inhibitor [thromboxane synthetase], Antithrombotic |
| 6.3.2.4 | D00877 | D-alanine-D-alanine ligase inhibitor, cell wall synthesis inhibitor | Antibacterial [tuberculostatic] |

**Table S8.** **Top prioritized enzymes (ParaNem).**

| **EC Num** | **Target**  **Score** | **Name** | **Type of enzyme** | **Previous Indications as Drug Target** |
| --- | --- | --- | --- | --- |
| 3.2.1.26 | 5 | β-fructofuranosidase | Hydrolase | -------------- |
| 1.14.19.3 | 2 | linoleoyl-CoA desaturase | Oxidoreductase | -------------- |
| 2.7.1.134 | 2 | inositol-tetrakisphosphate 1-kinase | Transferase | -------------- |
| 2.7.1.159 | 2 | inositol-1,3,4-trisphosphate 5/6-kinase | Transferase |  |
| 3.5.4.25 | 2 | GTP cyclohydrolase II | Hydrolase | Antimicrobial acitivity |
| 4.2.1.104 | 2 | Cyanase | Lyase | -------------- |
| 4.2.2.2 | 2 | pectate lyase | Lyase | Activity is associated with virulence in Pseudomonas viridiflava Virulence factor in N. hematococca (fungus); Disrupting a combination of proteins PL and pelD reduces virulence, but not proteins alone.  Found in plant nematodes. It is a cell wall degrading enzyme. Important for plant-parasite interaction. Found in Globodera rostochiensis (potato cyst nematode), B. mucronatus, etc. |

**Table S9. RNAseq reads stats and differential expressed genes.**

| **Sample** | **Control** | **Perhexiline (PER)** | **Etomoxir (ETO)** | **PER + ETO** | **Ivermectin (IVM)** |
| --- | --- | --- | --- | --- | --- |
| Putative Target | N/A | CPT1 and CPT2 | CPT1 | CPT1 and CPT2 | Glutamate channels |
| Cleaned reads # | 100,033,294 | 67,427,680 | 67,280,648 | 58,092,350 | 67,773,396 |
| Mapped reads # | 74,416,738 | 49,948,758 | 48,503,814 | 38,251,486 | 37,336,278 |
| Cleaned reads mapped % | 74.4% | 74.1% | 72.1% | 65.8% | 55.1% |
| Genes with transcript evidence # | 22,292 | 21,799 | 22,223 | 21,969 | 21,961 |
| Genes with transcript evidence % | 98.6% | 96.4% | 98.3% | 97.1% | 97.1% |
| Differentially expressed genes # | N/A | 2,094 | 1,595 | 1,966 | 2,869 |
| Differentially expressed genes % | N/A | 8.0% | 6.1% | 7.5% | 11.0% |
| Genes upregulated # | N/A | 747 | 744 | 455 | 1,016 |
| Genes upregulated % | N/A | 2.9% | 2.8% | 1.7% | 3.9% |
| Genes downregulated # | N/A | 1,347 | 851 | 1,511 | 1,853 |
| Genes downregulated % | N/A | 5.2% | 3.3% | 5.8% | 7.1% |

**Table S10. Gene Ontology enriched categories of upregulated *C. elegans* genes in presence of PER, ETO, PER+ETO and IVM.**

| **PER** | **ETO** | **PER & ETO** | **IVE** | **GO Term*** | **Total Number of Genes in Dataset** |
| --- | --- | --- | --- | --- | --- |
| Y | Y | Y | - | Biological Process: response to heat (GO:0009408) | 17 |
| Y | Y | Y | - | Cellular Component: peroxisome (GO:0005777) | 11 |
| Y | Y | - | - | Biological Process: multicellular organismal development (GO:0007275) | 30 |
| Y | Y | - | - | Molecular Function: structural constituent of cuticle (GO:0042302) | 160 |
| Y | - | Y | - | Biological Process: sex differentiation (GO:0007548) | 11 |
| Y | - | - | Y | Molecular Function: nucleotide binding (GO:0000166) | 314 |
| Y | - | - | - | Biological Process: small GTPase mediated signal transduction (GO:0007264) | 85 |
| Y | - | - | - | Molecular Function: GTP binding (GO:0005525) | 160 |
| - | Y | - | Y | Biological Process: Wnt receptor signaling pathway, calcium modulating pathway (GO:0007223) | 5 |
| - | Y | - | - | Biological Process: protein transport (GO:0015031) | 66 |
| - | Y | - | - | Cellular Component: extrinsic to membrane (GO:0019898) | 5 |
| - | Y | - | - | Cellular Component: proteinaceous extracellular matrix (GO:0005578) | 12 |
| - | Y | - | - | Molecular Function: serine-type carboxypeptidase activity (GO:0004185) | 9 |
| - | - | Y | - | Biological Process: fatty acid beta-oxidation (GO:0006635) | 9 |
| - | - | Y | - | Biological Process: fatty acid metabolic process (GO:0006631) | 16 |
| - | - | Y | - | Biological Process: glycerol metabolic process (GO:0006071) | 7 |
| - | - | Y | - | Cellular Component: myosin complex (GO:0016459) | 19 |
| - | - | Y | - | Molecular Function: 2 iron, 2 sulfur cluster binding (GO:0051537) | 11 |
| - | - | Y | - | Molecular Function: acyl-CoA dehydrogenase activity (GO:0003995) | 22 |
| - | - | Y | - | Molecular Function: acyl-CoA oxidase activity (GO:0003997) | 7 |
| - | - | Y | - | Molecular Function: flavin adenine dinucleotide binding (GO:0050660) | 59 |
| - | - | Y | - | Molecular Function: motor activity (GO:0003774) | 20 |
| - | - | Y | - | Molecular Function: oxidoreductase activity, acting on the CH-CH group of donors (GO:0016627) | 31 |
| - | - | - | Y | Biological Process: chromosome organization (GO:0051276) | 5 |
| - | - | - | Y | Biological Process: phospholipid transport (GO:0015914) | 6 |
| - | - | - | Y | Biological Process: regulation of transcription from RNA polymerase II promoter (GO:0006357) | 22 |
| - | - | - | Y | Biological Process: transcription initiation from RNA polymerase II promoter (GO:0006367) | 14 |
| - | - | - | Y | Cellular Component: cullin-RING ubiquitin ligase complex (GO:0031461) | 7 |
| - | - | - | Y | Cellular Component: intracellular (GO:0005622) | 679 |
| - | - | - | Y | Molecular Function: ATP binding (GO:0005524) | 890 |
| - | - | - | Y | Molecular Function: ATP-dependent helicase activity (GO:0008026) | 68 |
| - | - | - | Y | Molecular Function: DNA binding (GO:0003677) | 499 |
| - | - | - | Y | Molecular Function: exonuclease activity (GO:0004527) | 13 |
| - | - | - | Y | Molecular Function: helicase activity (GO:0004386) | 86 |
| - | - | - | Y | Molecular Function: nucleic acid binding (GO:0003676) | 517 |
| - | - | - | Y | Molecular Function: phospholipid-translocating ATPase activity (GO:0004012) | 6 |
| - | - | - | Y | Molecular Function: protein binding (GO:0005515) | 2352 |
| - | - | - | Y | Molecular Function: protein serine/threonine kinase activity (GO:0004674) | 233 |
| - | - | - | Y | Molecular Function: RNA binding (GO:0003723) | 146 |
| - | - | - | Y | Molecular Function: RNA polymerase II transcription factor activity (GO:0003702) | 19 |
| - | - | - | Y | Molecular Function: ubiquitin protein ligase binding (GO:0031625) | 7 |
| - | - | - | Y | Molecular Function: zinc ion binding (GO:0008270) | 1088 |
| *Y = Significant Enrichment among upregulated genes in gene set* , - = Not Significant. | | | | | |
