# Supplementary material for: Discovery of Anthelmintic Drug Targets and Drugs Using Chokepoints in Nematode Metabolic Pathways
Source: PLoS Pathog. 2013 Aug 1;9(8):e1003505. doi: 10.1371/journal.ppat.1003505 (PMC3731235; doi:10.1371/journal.ppat.1003505)
Supplement: Text S2 — Supporting figures. Figure S1. Chokepoints found in various groups. The total number of ECs that were mapped from AllKEGG and the number of those that are in KEGGChoke are shown in the two lighter grey colors. The number of chokepoint targets in the various groups that have ECs associated with a drug in KEGG Drug (dark grey) and DrugBank (black). CommNem, intersection of nematode ECs; UniNem, set of all nematode ECs; Hs, ECs from H. sapiens; Dm, ECs from D. melanogaster. Figure S2. Enriched and depleted enzyme categories based on EC nomenclature in various groups and species. Heatmap illustrating enrichment or depletion in the groups for A. chokepoint enzymes within KEGG (KEGGChoke) and B. enzymes from all of KEGG (AllKEGG). The extreme blue color indicates that the enzyme category was significantly depleted and the extreme red color indicates the enzyme category was significantly enriched relative to either KEGGChoke or AllKEGG using Fisher's Exact Test. The intermediate color shades indicate enrichment or depletion, but are not statistically significant. CommNem, intersection of ECs; UniNem, set of all nematode ECs; Hs, ECs from H. sapiens; Dm, ECs from D. melanogaster; DrugBank, ECs from DrugBank; KEGG Drug, ECs from KEGG Drug. Figure S3. Number of pathways in which an enzyme acts. The data is broken into enzymes acting in one pathway (light grey) versus multiple pathways (dark grey). Comparison of percentage of ECs involved in one versus multiple pathways in A. UniNem, CommNem, KEGGChoke, and AllKEGG and B. KEGG Drug, DrugBank, KEGGChoke, and AllKEGG. CommNem, intersection of ECs; UniNem, set of all nematode ECs; KEGGChoke, chokepoint enzymes within KEGG; AllKEGG, all enzymes within KEGG; DrugBank, ECs from DrugBank; KEGG Drug, ECs from KEGG Drug. (DOCX) [file ppat.1003505.s002.docx]

**SUPPORTING INFORATION**

**SUPPORTING FIGURES:**


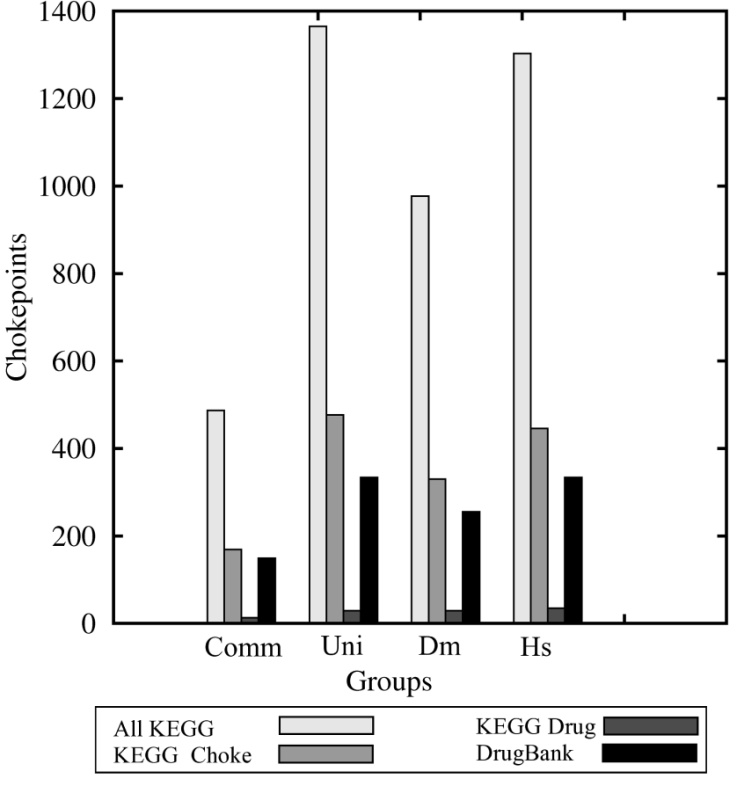


**Figure S1. Chokepoints found in various groups.** The total number of ECs that were mapped from AllKEGG and the number of those that are in KEGGChoke are shown in the two lighter grey colors. The number of chokepoint targets in the various groups that have ECs associated with a drug in KEGG Drug (dark grey) and DrugBank (black). Comm, intersection of ECs; Uni, set of all nematode ECs; Hs, ECs from *H. sapiens*; Dm, ECs from *D. melanogaster*.


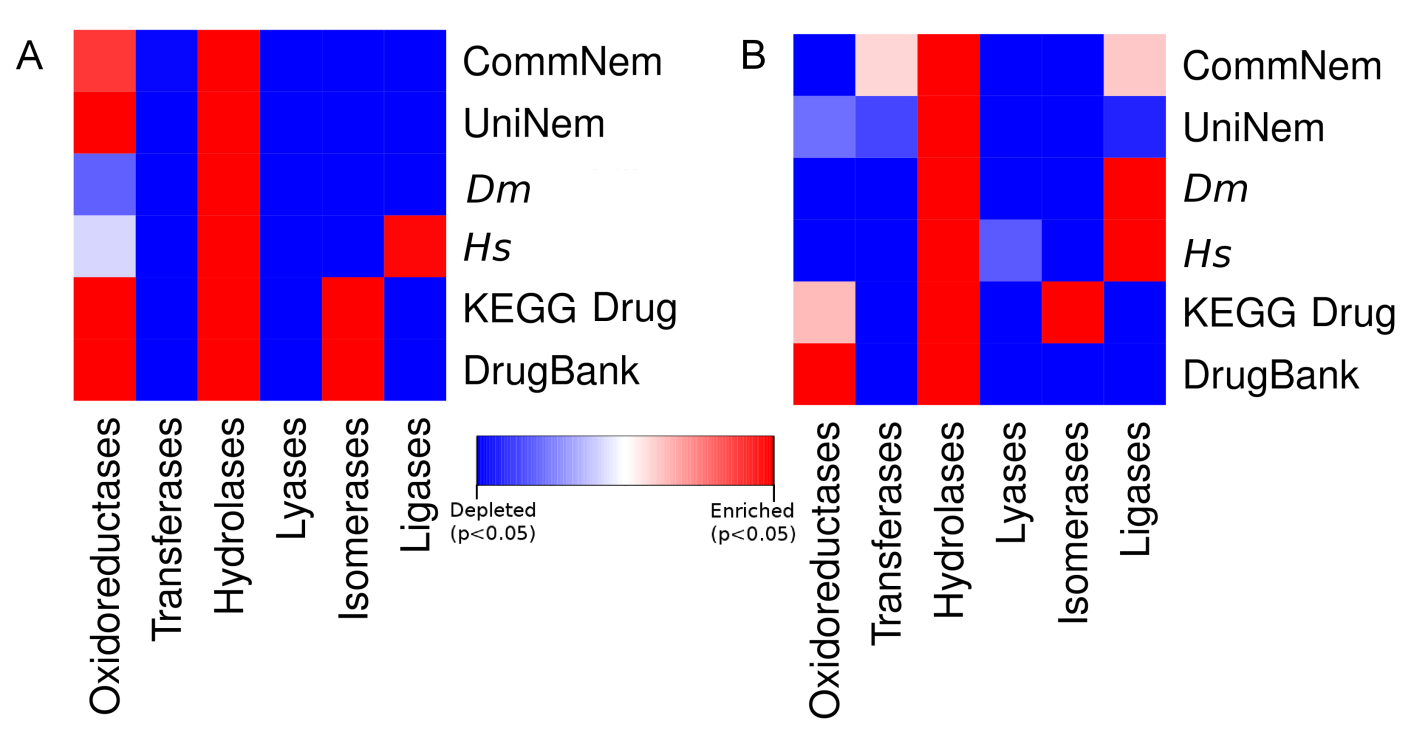


**Figure S2. Enriched and depleted enzyme categories based on EC nomenclature in various groups and species.** Heatmap illustrating enrichment or depletion in the groups for A. chokepoint enzymes within KEGG (KEGGChoke) and B. enzymes from all of KEGG (AllKEGG). The extreme blue color indicates that the enzyme category was significantly depleted and the extreme red color indicates the enzyme category was significantly enriched relative to either KEGGChoke or AllKEGG using Fisher’s Exact Test. The intermediate color shades indicate enrichment or depletion, but are not statistically significant. CommNem, intersection of ECs; UniNem, set of all nematode ECs; Hs, ECs from *H. sapiens*; Dm, ECs from *D. melanogaster*; DrugBank, ECs from DrugBank; KEGG Drug, ECs from KEGG Drug.


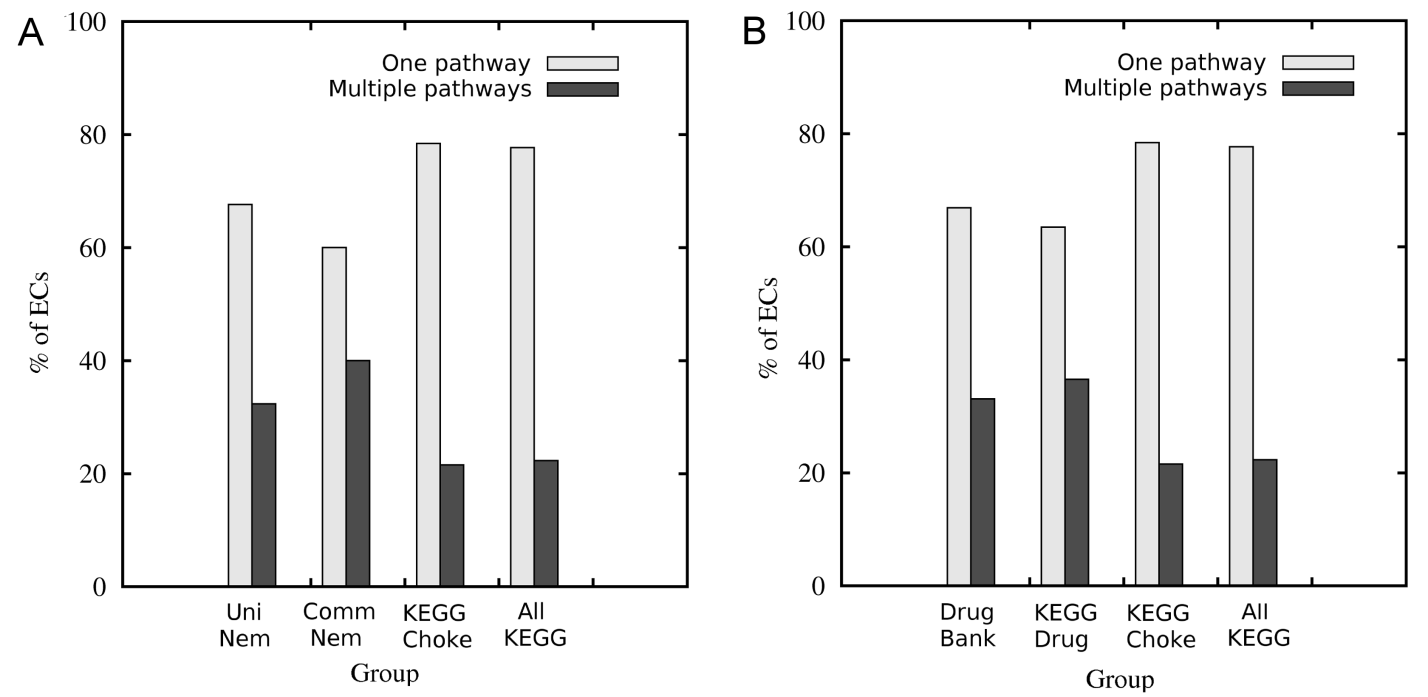


**Figure s3. Number of pathways in which an enzyme acts.** The data is broken into enzymes acting in one pathway (light grey) versus multiple pathways (dark grey). Comparison of percentage of ECs involved in one versus multiple pathways in A. UniNem, CommNem, KEGGChoke, and AllKEGG and B. KEGG Drug, DrugBank, KEGGChoke, and AllKEGG. CommNem, intersection of ECs; UniNem, set of all nematode ECs; KEGGChoke, chokepoint enzymes within KEGG; AllKEGG, all enzymes within KEGG; DrugBank, ECs from DrugBank; KEGG Drug, ECs from KEGG Drug.
